# APOL1 risk allele RNA contributes to renal toxicity by activating protein kinase R

**DOI:** 10.1038/s42003-018-0188-2

**Published:** 2018-11-07

**Authors:** Koji Okamoto, Jason W. Rausch, Hidefumi Wakashin, Yulong Fu, Joon-Yong Chung, Patrick D. Dummer, Myung K. Shin, Preeti Chandra, Kosuke Suzuki, Shashi Shrivastav, Avi Z. Rosenberg, Stephen M. Hewitt, Patricio E. Ray, Eisei Noiri, Stuart F. J. Le Grice, Maarten Hoek, Zhe Han, Cheryl A. Winkler, Jeffrey B. Kopp

**Affiliations:** 1 Kidney Disease Section, National Institute of Diabetes and Digestive and Kidney Diseases, National Institutes of Health, 9000 Rockville Pike, Bethesda, MD 20892 USA; 2 Division of Nephrology, Endocrinology and Vascular Medicine, Department of Medicine, Tohoku University Hospital, 1-1 Seiryo-machi, Aoba-ku, Sendai, Miyagi 980-8574 Japan; 3 Department of Nephrology, Endocrinology, Hemodialysis & Apheresis, University Hospital, The University of Tokyo, 7-3-1 Hongo, Bunkyo-ku, Tokyo, 133-8655 Japan; 4 Reverse Transcriptase Biochemistry Section, Basic Research Program, Frederick National Laboratory for Cancer Research, 1050 Boyle Street, Frederick, MD 21702 USA; 5 Children’s National Health System, 111 Michigan Ave NW, Washington, DC 20010 USA; 6 Experimental Pathology Lab, Laboratory of Pathology, Center for Cancer Research, National Cancer Institute, National Institutes of Health, 9000 Rockville Pike, Bethesda, MD 20892 USA; 7 Merck Research Laboratories, Merck and Co., Inc., 2000 Galloping Hill Rd, Kenilworth, NJ 07033 USA; 8 Division of Nephrology, Department of Medicine, University of Maryland School of Medicine, 655 W. Baltimore Street, Baltimore, MD 21201 USA; 9 Department of Pathology, Johns Hopkins Medical Institutions, 720 Rutland Avenue, Baltimore, MD 21287 USA; 10 Basic Research Laboratory, Center for Cancer Research, National Cancer Institute, Leidos Biomedical Research, Frederick National Laboratory, 8560 Progress Dr., Frederick, MD 21702 USA

## Abstract

*APOL1* risk alleles associate with chronic kidney disease in African Americans, but the mechanisms remain to be fully understood. We show that *APOL1* risk alleles activate protein kinase R (PKR) in cultured cells and transgenic mice. This effect is preserved when a premature stop codon is introduced to *APOL1* risk alleles, suggesting that *APOL1* RNA but not protein is required for the effect. Podocyte expression of *APOL1* risk allele RNA, but not protein, in transgenic mice induces glomerular injury and proteinuria. Structural analysis of the APOL1 RNA shows that the risk variants possess secondary structure serving as a scaffold for tandem PKR binding and activation. These findings provide a mechanism by which APOL1 variants damage podocytes and suggest novel therapeutic strategies.

## Abstract (web summary)

Okamoto et al. report that APOL1 RNA, but not protein, expressed from chronic kidney disease (CKD)-associated alleles activates protein kinase R, inducing glomerular injury in a mouse model. The secondary structure of APOL1 risk variant RNA acts as a scaffold for protein kinase R, suggesting potential therapeutic strategies for CKD.

## Introduction

African Americans have a four-fold increased lifetime risk for end-stage kidney disease^1^ compared to their non-African American counterparts. Apolipoprotein L1 (APOL1) is a component of the innate immune system, and APOL1 RNA expression is driven by interferon and tumor necrosis factor (TNF), key regulators of innate immunity^2–4^. APOL1 was first identified as a minor protein component of high density lipoprotein, and APOL1 circulating on plasma high density lipoprotein kills *Trypanosoma*, the cause of African sleeping sickness^5,6^. Population-based studies have shown that the *APOL1* variants G1 [rs73885319 AdeltaG (S342G) and rs60910145 TdeltaG (M384I)] especially rs73885319 AdeltaG (S342G)^7^ and G2 [rs71785313 TTATAA → del (NYK388K)] are strongly associated with increased risk for chronic kidney diseases in individuals of sub-Saharan African ancestry^8^. Kidney diseases most strongly associated with *APOL1* variants include HIV-associated nephropathy (reported odds ratio: 29–89^7,9^), focal segmental glomerulosclerosis (FSGS; reported odds ratio: 17^7^), and arterionephrosclerosis (hypertension-attributed kidney disease; reported odds ratio: 7) for progression to end-stage kidney disease^10^. The *APOL1* G1 and G2 protein isoforms lyse *Trypanosoma brucei rhodesiense* that are resistant to the ancestral G0 variant, which may explain why these variants have risen to high prevalence in sub-Saharan African populations^11^.

The most strongly APOL1 variant-associated diseases, FSGS and HIV-associated nephropathy, involve injury to podocytes, which express high levels of APOL1^12^. In transgenic mice, expression of human risk variant APOL1 in podocytes leads to FSGS^13^. Several mechanisms of *APOL1* risk variant-induced cell injury have been suggested, including transcellular cation flux^14^, mitochondrial dysfunction^15^, and dysfunction of endosomal trafficking^16,17^. However, there may be additional mechanisms by which APOL1 variants injure podocytes.

In the present work, we investigated a novel mechanism linking *APOL1* genetic variants to podocyte injury. We present evidence that the APOL1 G1 and G2 variant RNAs contribute to cell injury. The APOL1 disease-associated variants encode RNAs that are predicted to form longer regions of double-stranded RNA, compared to the APOL1 G0 variant RNA. Double-stranded RNA activates protein kinase R (PKR), which turn phosphorylates eukaryotic translation initiation factor 2A (EIF2α). eIF2α is a key regulator of protein synthesis and phosphorylated eIF2α (the inactive form) inhibits translation initiation^18^. Translation inhibition is an alarm signal and a defense mechanism that forms part of the innate immune response^19^. Further, in a transgenic rat model, inhibition of protein translation was associated with glomerulosclerosis^20^. This new finding regarding PKR expands the list of mechanisms by which *APOL1* variants damage podocytes^21^ and provides a novel therapeutic target.

## Results

### APOL1 risk variants inactivated eIF2-α via PKR activation

**Fig. 1:**
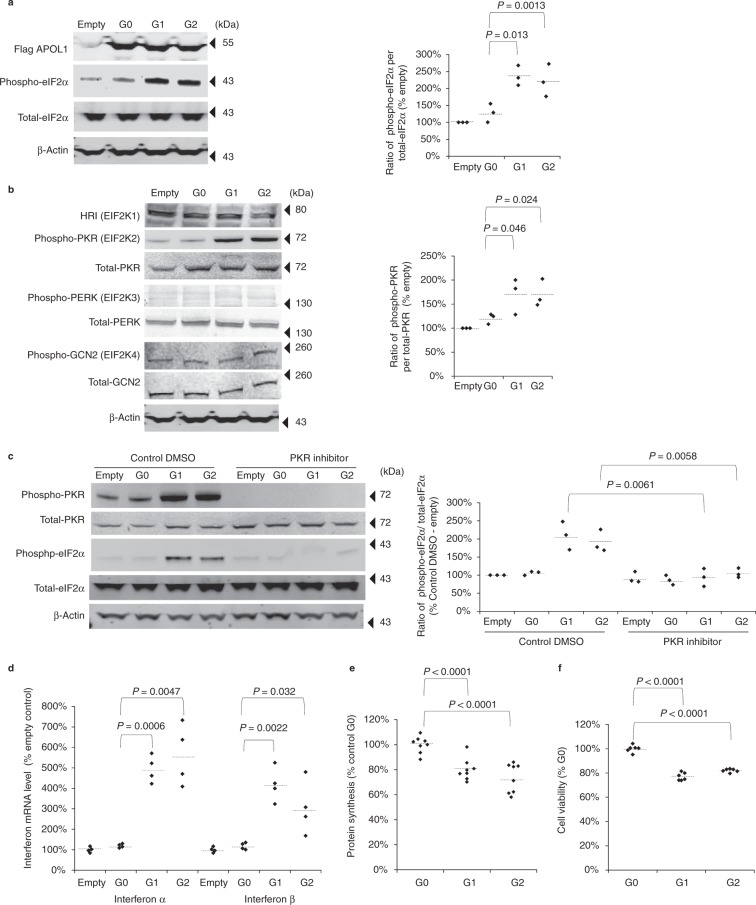
APOL1 risk variants activated PKR and downstream signals. **a** HEK293FT cells over-expressing APOL1 were harvested for detection of APOL1 and eIF2α protein by Western blotting. Renal risk variants G1 and G2 increased phospho-eIF2α compared to the G0 allele. **b** HEK293FT cells over-expressing APOL1 were harvested to identify which of the four eIF2 kinases (HRI, PKR, PERK, GCN2, respectively) were activated. The G0 variant showed minimally increased phospho-PKR, indicating activation, while renal risk variants G1 and G2 manifested substantially increased phospho-PKR. **c** HEK293FT cells over-expressing APOL1 were treated with a PKR inhibitor or vehicle control, then harvested for detection of PKR and eIF2α. The APOL1 renal risk variants increased phospho-PKR and phospho-eIF2α compared to the G0 allele, effects that were abolished or greatly diminished by PKR inhibition. **d** Transfected HEK293FT cells expressing APOL1 RNA encoded by the G1 and G2 variants increased interferon α and interferon β RNA expression compared to the G0 variant, as measured by qRT-PCR. **e** Total protein synthesis was analyzed in transfected HEK293FT cells expressing APOL1 RNA with the G0, G1, and G2 variants. Translation initiation was measured using the L-azidohomoalanine protein synthesis assay. Quantification of protein synthesis, with the APOL1 risk variants measured against the G0 transfected cells (set to 100%) and puromycin exposure (set to 0%). Both risk variants G1 and G2 reduced protein synthesis by HEK293FT cells. **f** Quantification of cell viability of stable HEK293 cell line expressing APOL1. The cells were lysed and incubated with CellTiter-Glo to measure the amount of ATP in the cells. HEK293 cells with the G1 and G2 variants manifested decreased cell viability compared with the G0 variants. Each dot is calculated from one well. Each horizontal line represents mean. All results are presented as ratio of controls, normalized to 100% and *P* values were calculated using a Student one-tailed *t*-test. Welch correction was used for panel (**d**)

We hypothesized that *APOL1* genetic variants might interfere with translation initiation, and therefore we examined the status of eIF2α. We found markedly increased phosphorylation in HEK293FT cells that over-expressed *APOL1* risk alleles G1 or G2 compared to wild type G0 allele or empty vector (Fig. 1a). Plasmid information is available in Supplementary Figure 1.

eIF2α is inactivated by four kinases including the general control non-derepressible 2 kinase (GCN2), PKR-like endoplasmic reticulum kinase (PERK), interferon-induced, double-stranded RNA-activated protein kinase (PKR), and heme-regulated inhibitor kinase (HRI)^22^. We examined the activation status of these four kinases. For further analysis, HEK293FT cells were pre-incubated with interferon α as previously reported^23^. Only pPKR, the activated form, was increased by *APOL1* risk alleles (Fig. 1b and Supplementary Figure 2a). In addition, we found that phosphorylated eIF2α was decreased in the presence of a PKR-specific inhibitor (Fig. 1c), further implicating PKR as the mediator.

### APOL1 variants induce interferon and inhibit protein synthesis

PKR also activates IκB alpha kinase (IKK) and induces an interferon response^24^, consistent with our observation that cells expressing *APOL1* risk alleles G1 and G2 induced type 1 interferons (Fig. 1d). Global protein synthesis and cell viability were also reduced, respectively, relative to G0, in agreement with known effects of eIF2α phosphorylation (Fig. 1e, f). Together, these results suggest that *APOL1* risk alleles activate PKR, which in turn produces an interferon response, leading to eIF2α phosphorylation to inhibit protein synthesis and cause toxic effect on cells.

### APOL1 risk variant mRNA activates PKR

**Fig. 2:**
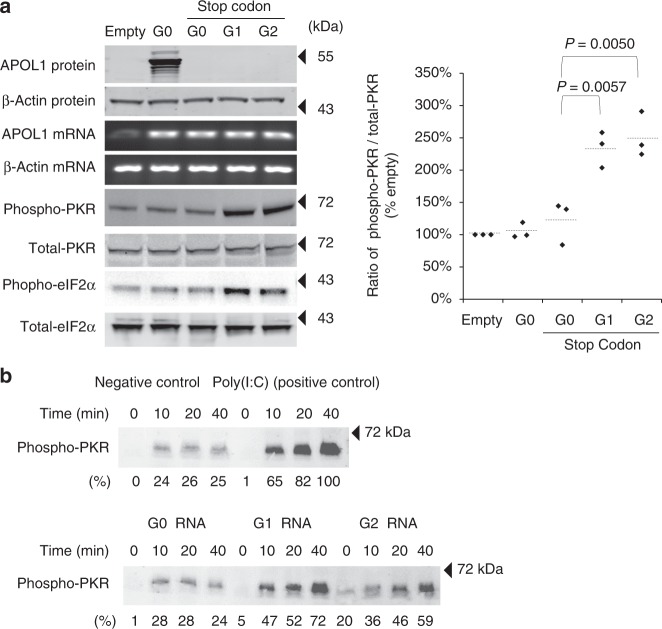
APOL1 RNAs from high-risk alleles activate PKR. **a** Stable HEK293FT cell lines expressing APOL1 RNA (but not protein) were harvested for detection of APOL1 and β-actin mRNAs and eIF2α and PKR proteins. Constructs expressing APOL1 risk variant G1 and G2 RNA (without protein) increased phospho-PKR and phospho-eIF2 alpha, whereas the G0 variant had no effect. **b** APOL1 G1 and G2 RNA promote PKR activation in a time-dependent manner. RNAs (NM_001136540.1, 298–1453) transcribed in vitro using T7 sequence were incubated with PKR and ATP for indicated times. Poly(I:C) served as the positive control for PKR phosphorylation. Vehicle was used for negative control. Western blots targeting phospho-PKR are presented. The quantified intensity of phospho-PKR to each RNAs was added below the gel images

PKR is activated by double stranded RNA (dsRNA)^25^. When two molecules of PKR bind to dsRNA structure in a tandem fashion, PKR autophosphorylation occurs, leading to functional activation. To test whether APOL1 mRNA is sufficient for PKR activation, we created APOL1 RNA expression vectors engineered to contain premature stop codons that allow expression of full-length mRNA but prevent translation of APOL1 protein. Using these vectors, we demonstrated that APOL1 risk allele mRNA alone is sufficient to increase levels of phosphorylated PKR in stably-transfected cell lines (Fig. 2a). RNA immunoprecipitation using antibody against phosphorylated-PKR detected more G1 and G2 mRNA compared with G0 mRNA, suggesting that the disease-associated variant RNAs were more tightly bound to PKR (Supplementary Figure 2b). To exclude the possibility that PKR is activated by a collateral pathway^26,27^ or by a truncated protein generated by alternative start-codon or differential splicing, we performed in vitro experiments using only synthesized APOL1 RNA, purified recombinant PKR protein, and ATP. These experiments confirmed that isolated APOL1 risk allele RNA independently promotes PKR autophosphorylation (Fig. 2b and Supplementary Figure 2c).

### APOL1 risk variant mRNAs have more stable dsRNA structures

To investigate the sequence and structural requirements for this activity, we divided full-length APOL-G1 RNA into four sections (average length ~319 nt) and conducted PKR activation assays. Of the four RNAs, only the truncated APOL1 RNA (NM_001136540: 1180–1453), containing the polymorphic regions of the *APOL1* gene and a small segment of the 3′ untranslated region (UTR), was sufficient to activate PKR (Supplementary Figure 2d). As with the full-length transcripts, truncated APOL1 RNA was sufficient for risk variants to activate PKR compared to the G0 truncated APOL1 RNA (Supplementary Figure 2e).

**Fig. 3:**
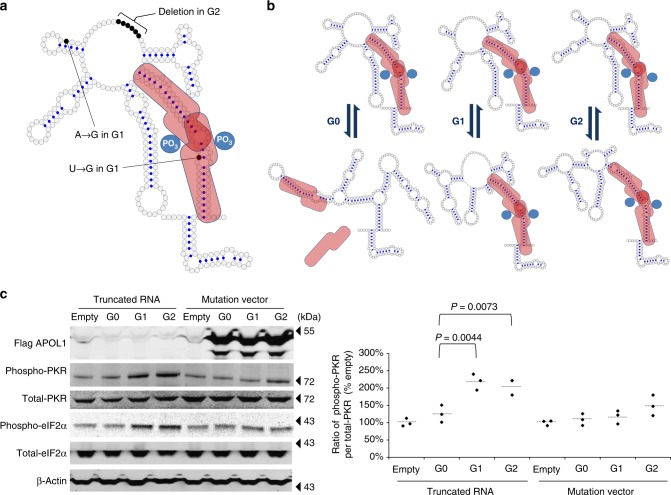
APOL1 RNA secondary structure serving as a scaffold for tandem PKR binding. **a** We generated lowest-energy secondary structural models for the truncated APOL1 G0 RNA variant (NM_001136540.1, 1180–1453) using RNAstructure software with SHAPE-derived reactivity profiles. Sequence differences between G0 and the G1 and G2 RNA variants are indicated here for convenience, although it should be noted that G1 and G2 variants do not occur on the same chromosome. A bipartite PKR is depicted in red to reflect the presence of distinct RNA binding and kinase domains in each protein monomer. Two PKR molecules are known to bind in tandem to long RNA duplexes, and this binding promotes autophosphorylation of PKR kinase domains and increases kinase activity. Blue dots mark sites at which Watson–Crick (G–C or A–T) or non-canonical (G–U) base pairing are predicted. We propose that the ~33 bp interrupted duplex motif within this segment of APOL1 RNA may serve as a docking site for tandem PKR binding. **b** Structure-based equilibrium model for APOL1 RNA-mediated PKR activation. SHAPE-derived secondary structural models of lowest energy (top) and second-lowest energy (bottom), along with their proposed interactions with PKR, are depicted. SHAPE and non-denaturing polyacrylamide gel electrophoresis indicate that APOL1 RNAs can assume alternative low energy conformations that may exist in a dynamic equilibrium. For APOL1 G0, the second-lowest energy model structure lacks a second PKR docking site, effectively reducing the number of such sites available for PKR binding autophosphorylation in a heterogeneous mixture of G0 RNA conformers and thus reducing PKR activation. This is in contrast to the proposed equilibrium states of the G1 and G2 RNAs, wherein both low energy conformers contain PKR docking sites and would therefore be expected to support PKR activation to a greater extent. **c** We generated stably-transfected HEK293FT cell lines expressing truncated APOL1 RNA which contain the APOL1 G0, G1, or G2 allele together with mutated variants whose secondary structure is disrupted by eight synonymous mutations. The mutated RNAs failed to promote PKR phosphorylation. All results are presented as ratio of empty, normalized to 100%. *P* values were calculated using a Student one-tailed *t*-test. Each horizontal line represents mean

Secondary structures for these RNA constructs were determined using selective 2′-hydroxyl acylation analyzed by primer extension (SHAPE). The lowest-energy (stable) structure predicted for truncated G0 APOL1 RNA is depicted in Fig. 3a. APOL1 G0/G1/G2 RNAs all contain a ~33 bp duplex region that is long enough to be a docking site for tandem PKR binding and PKR autophosphorylation^28^. Specifically, of the two lowest-energy structures predicted for each variant, only G0 RNA lacks a PKR docking site (Fig. 3b, Supplementary Figure 3 and Supplementary Figure 4a). These data suggest that a PKR docking site would likely be more readily available within G1 and G2 RNA compared to G0 RNA.

Cell lines stably expressing truncated APOL1 RNA sequences derived from the G1 and G2 variants, but not the G0 variant, demonstrated increased PKR activation (Fig. 3c). Moreover, introducing eight synonymous mutations into these RNA constructs completely altered the structures of all three APOL1 alleles (Supplementary Figure 3), rendering them incapable of supporting PKR autophosphorylation (Fig. 3c) and preventing cell death (Supplementary Figure 3b). Conversely, we engineered synonymous mutations in the APOL1 G0 RNA sequences that were predicted to increase dsRNA stability and found that this modified G0 RNA activated PKR (Supplementary Figure 4c and 4d). These data further support our postulate that APOL1 RNA-mediated PKR activation is dependent on RNA secondary structure.

### Confirmation with human kidney and podocyte cell lines

**Fig. 4:**
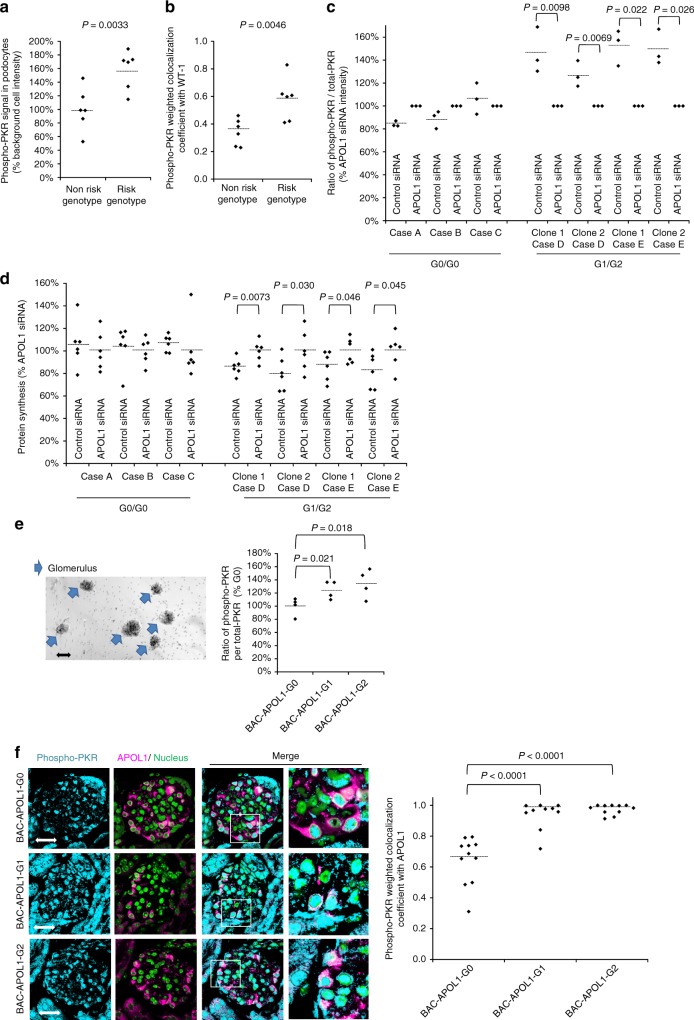
APOL1 risk variant activated PKR in human kidney tissue podocytes, in cultured human podocytes, and human APOL1 gene locus transgenic mice (BAC-APOL1 mice). **a** Kidney tissue from subjects with glomerular disease tissue was assessed for phospho-PKR; signal intensity was calculated, with average intensity in non-risk genotype cases set to 100%. Each dot represents the average signal intensity from one case. **b** Quantification of weighted colocalization coefficient between phospho-PKR and WT-1 revealed an elevated phospho-PKR in podocytes of risk genotype individuals. **c** Conditionally immortalized human podocytes, one cell line from each of three APOL1 G0/G0 FSGS patients and two cell lines from each of two APOL1 G1/G2 FSGS subjects, were transfected with APOL1 siRNA or control siRNA. The ratio of phospho-PKR signal to total-PKR was measured from Western blot. Knock down efficiencies were 52.4–75.4% (RNA levels) or 31.1% (protein levels). G1/G2 podocytes manifested increased PKR phosphorylation, which was diminished by APOL1 RNA knock-down (*n* = 3). Raw values are in Supplementary Table 2. **d** Quantification of protein synthesis in conditionally immortalized human podocytes after 96 h transfection with APOL1 siRNA or control siRNA. Protein synthesis was reduced in G1/G2 cases compared to G0/G0 cases (*n* = 6). Raw values are in Supplementary Table 3. **e** Glomeruli were isolated from BAC-APOL1 transgenic mice using magnetic particles (upper panel: arrows indicated glomeruli, small dots are magnet beads). Bar represents 50 μm. Glomeruli were treated with phosphatase inhibitor with/without PKR inhibitor for 30 min and lysed for Western blot analysis. Phospho-PKR was increased in G1 and G2 glomeruli. **f** Immunofluorescence staining was visualized using confocal microscopy. Phospho-PKR was visualized as turquoise blue, APOL1 as magenta, and nucleus was as green. The increased number of yellow cells in the BAC-APOL1-G1 and G2 mouse overlay image suggests PKR activation (phosphorylation) in podocytes. Bar represents 20 μm. Quantification of weighted colocalization coefficient between phospho-PKR and APOL1 revealed an elevated phospho-PKR in podocytes of the BAC-APOL1-G1 and G2 mice. Each dot represents a glomerulus. Each horizontal line represents the mean. *P* values were calculated using a Student one-tailed *t*-test

In formalin-fixed, paraffin-embedded kidney tissue obtained from patients with FSGS, phosphorylated PKR was increased in glomeruli from subjects with the two *APOL1* risk alleles (Fig. 4a, b, Supplementary Figure 5a and b). We also conducted knock-down experiments using conditionally immortalized human cell lines established from human urine. In APOL1 G1/G2 podocyte clones but not in G0/G0 podocyte clones, knock down of APOL1 RNA reduced PKR phosphorylation (Fig. 4c and Supplementary Figure 5c) and increased protein synthesis (Fig. 4d).

### Human *APOL1* gene locus mice and APOL1 RNA-only mice

Because APOL1 is unique to humans and some primates^29^, we used transgenic mice using the human *APOL1* gene locus contained within a bacterial artificial chromosome (BAC-APOL1 mice; provided by M. Hoek, Merck and Company) (Supplementary Figure 6a). Using these mice, we found that the phosphorylated PKR signal was increased in the BAC-APOL1-G1 and BAC-APOL1-G2 mice compared to BAC-APOL1-G0 and wild-type mice lacking the *APOL1* gene (Fig. 4e, f and Supplementary Figure 6b).

**Fig. 5:**
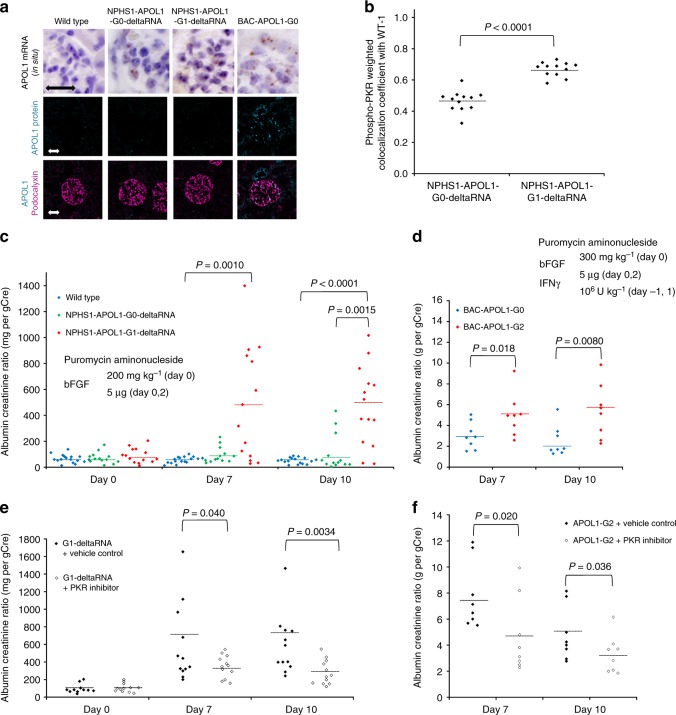
APOL1 risk allele RNA increased PKR activation and proteinuria in transgenic mice. **a** Transgenic mice were generated with a NPHS1 promoter driving APOL1 truncated RNA. RNA was expressed in glomerular cells from all APOL1 mice, both those expressing truncated RNA mice and the BAC-APOL1 mice, as shown by in situ hybridization. APOL1 protein was present only in the BAC-APOL1 mice, as expected. APOL1 was visualized as turquoise blue and podocalyxin was as magenta. Bar represents 20 μm. **b** Quantification of weighted colocalization coefficient between phospho-PKR and APOL1 revealed an elevated phospho-PKR in podocytes of the NPHS1-APOL1-G1-deltaRNA mouse. A single dot represents data from one glomerulus. **c** NPHS1-APOL1-deltaRNA transgenic mice manifested more proteinuria following podocyte injury after initiation of puromycin aminonucleoside and basic FGF exposure, assessed as albumin per creatinine ratio (mg per g), with higher levels in NPHS1-APOL1-G1-deltaRNA transgenic mice compared to NPHS1-APOL1-G0-deltaRNA transgenic mice. Urine protein was measured on days 0, 7, and 10 after initiation of puromycin aminonucleoside and basic FGF exposure, which induce podocyte injury. Each value represents data from one mouse. **d** BAC-APOL1 transgenic mice manifested more proteinuria following podocyte injury after initiation of interferon γ, puromycin aminonucleoside, and basic FGF, assessed as urine albumin/creatinine ratio (g per g), with higher levels in BAC-APOL1-G2 transgenic mice compared to BAC-APOL1-G0 transgenic mice. Urine protein was measured on days 7 and 10 after initiation of puromycin aminonucleoside, basic FGF exposure, and IFNγ, which induce podocyte injury. Each value represents data from one mouse. **e** NPHS1-APOL1-G1-delta-RNA transgenic mice received the PKR inhibitor or vehicle. Proteinuria, assessed as urine albumin per creatinine ratio (mg/g), was significantly reduced at days 7 and 10 after podocyte injury induced by puromycin aminonucleoside plus basic FGF induction. **f** BAC-APOL1-G2 transgenic mice received the PKR inhibitor or vehicle. Proteinuria was assessed as urine albumin per creatinine ratio (g/g) and was significantly reduced by the PKR inhibitor at days 7 and 10 after puromycin aminonucleoside plus basic FGF plus IFNγ induction of podocyte injury. Each value is from one mouse. Each horizontal line represents mean. *P* values were calculated using a Wilcoxon one-tailed *t*-test (**c**). *P* values were calculated using a Student one-tailed *t*-test (**b**, **d**–**f**)

We also produced transgenic mice expressing, in a podocyte-specific fashion, truncated mRNA around the APOL1 risk polymorphisms (NM_001136540, 1031–1453); these are termed NPHS1-APOL1-G0-delta-RNA mice and NPHS1-APOL1-G1-delta-RNA mice (Supplementary Figure 6c). In glomeruli from these mice, truncated APOL1 mRNA was transcribed in podocytes while, as expected, APOL1 protein expression was lacking (Fig. 5a and Supplementary Figure 6d). The amount of phosphorylated PKR was increased in NPHS1-APOL1-G1-delta-RNA mice compared to wild-type or NPHS1-APOL1-G0-delta-RNA mice (Fig. 5b). These two APOL1 transgenic mouse lines did not manifest albuminuria or glomerulosclerosis during the course of the study (Supplementary Figure 6e and Fig. 5c). We induced podocyte injury in mice by administering basic fibroblast growth factor (bFGF) and puromycin aminonucleoside, which produce FSGS^30^. In this model (NPHS1-APOL1-G1-delta-RNA mice) (Fig. 5c) and in BAC-APOL1-G2 mice (Fig. 5d), mice exhibited increased albuminuria at days 7 and 10. We injected the specific PKR inhibitor C16 (3.35 μg per kg body weight) daily beginning at day 0 and demonstrated that PKR inhibitor reduced phospho-PKR (Supplementary Figure 6f). Albuminuria decreased in PKR inhibitor-treated mice on days 7 and 10, lending support for a role of PKR activation in podocyte injury (Fig. 5e, f).

## Discussion

We report that *APOL1* genetic variants that are strongly associated with kidney disease among African descent individuals activate PKR and this contributes to podocyte injury in vitro and in vivo. BAC-APOL1 mice strains express APOL1 mRNA under the control of the native human APOL1 promoter and likely other regulatory elements. In contrast to transgenic mice in which APOL1 is expressed from a doxycycline-inducible *NPHS1* promoter^13^, BAC-APOL1 mice do not exhibit a spontaneous kidney phenotype, as was the case for mice bearing a non-inducible NPH1 promoter^31^. Similarly, most individuals with two *APOL1* risk alleles do not develop kidney disease. In human transcriptome data from subjects with glomerular disease, the glomeruli from APOL1 high-risk subjects, compared to APOL1 low-risk subjects, manifested alterations in glomerular gene expression that were mapped to interferon and NF-kB pathways, both of which lie downstream of activated PKR^32^ HEK293FT over-expressing cell also needed interferon pre-treatment to induce sufficient PKR protein for PKR activation on the target RNA (Supplementary Figure 7a)^23^. If PKR is continuously active, this would promote excessive innate immune system activation. Therefore, it appears that two hits are required to confer podocyte stress: APOL1 variants and another factor or factors, such as interferon or viral infection.

Most studies have shown that the effect of APOL1 risk variants requires two risk alleles, although a single copy of the G1 risk allele slightly increases the risk for HIV-associated nephropathy^7,8^. Our results using an in vitro overexpression approach also fit a two risk allele model (Supplementary Figure 7b). It is possible that a dominant negative effect by G0 or quantitative threshold of toxicity from G1 or G2 could explain these data. To address this issue, we produced G0/G2 heterozygous mice from BAC-APOL1-G0 heterozygous and G2 heterozygous parents (Supplementary Figure 8a). According to the mouse serum APOL1 levels, it appears that both the APOL1-G0 and APOL1-G2 genes in the double transgenic mice transcribed APOL1 to the same extent as APOL1-G0 single transgene mice or APOL1-G2 single transgene mice (Supplementary Figure 8b). In the nephrotic model induced by puromycin and FGF, a dominant negative effect of APOL1-G0 was not observed (Supplementary Figure 8c). Thus, we concluded that APOL1-G0 does not abolish the toxic effect of APOL1-G2, but rather the difference between the effect of one risk allele and two risk alleles arises from the quantitative effect of two risk alleles. Further, in human genetic studies, the upstream APOL1-G1 SNP, rs73885319 A delta G (S342G), without the downstream G1 SNP, is sufficient to convey increased kidney disease risk^7^ and our in vitro data are concordant with this finding (Supplementary Figure 9).

In the SHAPE results, APOL1 G0 mRNA was predicted to contain a ~33 bp duplex region. No other motif in this RNA conformer meets the minimum structural requirements established for PKR activation^28^. However, all three APOL1 RNA variants are structurally heterogeneous (Supplementary Figure 10). Given a structurally heterogeneous and/or equilibrium condition among APOL1 RNA conformers, we propose that a PKR docking site would likely be more readily available to support PKR activation in a population of high-risk allele RNA compared to low risk alleles. Also, APOL1 RNA has a cis-bulge structure that has been previously reported in other RNAs to promote PKR binding^33^.

It is known that mRNAs possess functions beyond protein encoding. For example, the 3′ (UTR) of the brain-derived neurotrophic factor transcript stabilizes the RNA and extends its half-life^34^. The 3′ UTR of human *TNFA* gene contains a stem loop structure that activates PKR, activating eIF2α, which in turn increases the efficiency with which *TNFA* mRNA is spliced^35^. Notably the mRNA encoding two components of innate immunity, APOL1 and TNF, both activate PKR, which is itself a participant in innate immune defense.

The relative contributions of APOL1 protein and APOL1 RNA to APOL1 nephropathy remain uncertain. The results of the PKR inhibitor experiment involving a podocyte cell line (Supplementary Figure 11) and full-length APOL1 transgenic mice (Fig. 4f) suggests that the PKR pathway contributes significantly to renal toxicity. Specifically, the level of albuminuria of APOLl-G2 mice treated with PKR inhibitor was as same level as that of APOL1-G0 mice. This suggests that PKR activation may arise from APOL1 RNA effects and additional toxic effects may arise from APOL1 protein, although the mechanism for the latter is not clear. PKR activates interferon transcription (Fig. 1d), which in turn increases APOL1 transcription^36,37^; interferon also increases the transcription of PKR (Supplementary Figure 7a)^23^. This potential positive feedback loop is controlled by the limited supply of RNA capable of binding PKR.

Multiple mechanisms that have been proposed to account for APOL1 induced cell injury, including ion channel dysfunction, mitochondrial dysfunction, endolysosomal dysfunction, integrin activation, and inflammasome activation^13,15,17^. With regard to mitochondrial function, we found that a PKR inhibitor reduced oxygen consumption rate in G1/G2 podocytes but not in G0/G0 podocytes (Supplementary Figure 12). To determine the relative contribution of APOL1 RNA and APOL1 protein to cell injury, further studies will be needed using multiple models. Taken together, the data suggest that APOL1 protein and RNA function synergistically to promote the podocyte injury.

An unanswered question is why the APOL1 risk variant phenotype is limited to the kidney. PKR is a ubiquitous protein present in many cells. Podocytes, macrophages, and brain neurons express an abundance of both APOL1 and PKR. This may explain in part why glomerular disease is the most notable manifestation of *APOL1* risk variants. Activated PKR was present in cell culture and mouse models presented here, and this also occurs in other neural and hematopoietic diseases^38–41^. These observations support the concept that PKR levels are important for APOL1 toxicity and a PKR inhibitor is a possible therapeutic approach.

In conclusion, our results link expression of high-risk APOL1 RNA allelic variants with PKR activation, providing a mechanism by which *APOL1* risk variants contribute to podocyte injury. Targeting APOL1, PKR or other components of pathways downstream of activated PKR opens novel therapeutic approaches to treating APOL1-associated nephropathy.

## Methods

### Cell culture

Human embryonic kidney 293FT (HEK293) cells were grown in DMEM supplemented with 10% FBS and 1% penicillin/streptomycin (100 iU per ml; 100 μg per ml). Cell lines were not authenticated. Cells were free of mycoplasma. The cells were incubated in a humidified atmosphere of 5% CO_2_ at 37 °C. For stable cell lines, transfected cells were selected in the presence of puromycin (3 μg per ml). For transient transfection studies, HEK293 cells were seeded into 6-well plates in the presence of the vectors using Lipofectamin2000 (ThermoFisher). Podocyte cell lines were established from human urine^42^ and APOL1 genotyping was performed, after informed consent was obtained under research protocols (94-DK-0127, 94-DK-0133) approved in advance by the NIDDK Institutional Review Board. Case A is G0/G0 male with FSGS, case B is G0/G0 male with HIV-associated FSGS, case C is G0/G0 male with FSGS (HP55-345O-1), case D is G1/G2 male with FSGS, and case E is G1/G2 male with HIV-associated FSGS. Cells were grown in RPMI1640 supplemented with 10% FBS, ITS, and 1% penicillin/streptomycin (100 iU per ml; 100 μg per ml). Cells were incubated in a humidified atmosphere of 5% CO_2_ at 33 °C and differentiated at 37 °C for 7–10 days. Cell lines were not authenticated. Cells were free of *Mycoplasma*. Prior to collecting the cell lysates, cells were treated with 10^3^ U per ml IFNα (Abcam) for 24–72 h and 100 mM calyculin A (LC Laboratories) for 30 min. For PKR inhibitory experiments, cells were treated with 1 μM imidazolo-oxindole PKR inhibitor C16 (Sigma Aldrich) for 1 h^43^.

### Human tissue

Deidentified human kidney biopsy tissues were obtained from Dr. Preeti Chandra, University of Maryland. The research was approved in advance by the Institutional Review Boards at the University of Maryland and NIDDK, NIH.

### Plasmid information

The APOL1 G0 expression vector consists of CMV promoter derived human APOL1 cDNA (NM_001136540: 298–1453). For APOL1 G1 (rs73885319 A→G and/or rs60910145 T→G) and G2 (rs71785313 del), site-directed mutagenesis was performed to make it. For APOL1 expression vector lacking protein expression was produced by inserting a stop codon and frameshift sequence (TAATAGATGA) after the 138th codon. For the secondary structure mutation vector, original sequences are altered by eight synonymous changes (NM_001136540: 1195A→T, 1196G→C, 1197C→G, 1203A→T, 1209G→A, 1221G→A, 1224C→G, and 1242T→C). For stable dsRNA G0 vector original sequences are altered by 5 mutations in 3′ UTR (CCA CAG GGC AGG GCA GCC ACC AGG AGA GAT ATG CCT GGC AGG GGC CAG G→CCA CAG GGC AGG **C**CA GCC ACC A**A**G A**A**A GAT ATG C**T**T G**A**C AGG GGC CAG G). For flanking RNA around the APOL1 alleles (NM_001136540.1, 1031–1453), pRNAT-CMV3.2/Puro (GeneScript) was used as a background vector. Schemes of vector maps are in Supplementary Figure 1.

### Immunoblotting

Cells were lysed in a RIPA buffer contains protease inhibitor/phosphatase inhibitor cocktail. Lysates were separated by SDS-polyacrylamide gel electrophoresis and the proteins subjected to western blotting and blocked for 30 min in Odyssey blocking buffer (LI-COR). Blots were incubated with primary antibodies against APOL1 (Sigma Aldrich HPA018885), phospho-eIF2α (Cell Signaling Technology #9721), eIF2α (Cell Signaling Technology #5324), β-Actin (Santa Cruz Biotechnology Inc. sc-47778), HRI (Upstate #07-728), phospho-PKR (Santa Cruz Biotechnology Inc. sc-101784), PKR (abcam ab45427), phospho-PERK (Cell Signaling Technology #3179), PERK (Cell Signaling Technology #5683), phospho-GCN (Abcam ab75836), and GCN (Cell Signaling Technology #3302), LC3 (Abcam ab168803) and p62 (Cell signaling #5114). Blots were incubated with dye-labeled anti-rabbit antibody (LI-COR). All blots were imaged using the Odyssey infrared scanner (LI-COR). Raw gel images are shown in Supplementary Figure 13.

### Reverse transcriptase PCR and quantitative real-time PCR

Cells or total kidney were harvested in TRIzol reagent and total RNA was isolated. Total RNA was treated with DNase prior to synthesis of cDNA with oligo (dT). One 5-μg aliquot of RNA was used for cDNA synthesis by Superscript II reverse transcriptase. Samples were analyzed by PCR (RT) or quantitative RT-PCR (qRT-PCR) using Power SYBR Green PCR master mix (ThermoFisher). Relative expression in each sample was calculated as a ratio (attamoles specific gene per β-actin). Primer pairs are listed in Supplementary Table 1.

### Protein synthesis assay

Transfected HEK293 cells or human podocyte cell lines were grown in 96-well plates and were treated with 10^3^ U per ml IFNα (Abcam) for quantification or chamber slide for visualization. The synthesis assay (Click-iT AHA Alexa Fluor 488 Protein Synthesis HCS Assay, catalog no. C10289, Invitrogen) was carried out per manufacturer’s instruction. Nuclear counter stain was performed with Hoechst. HEK293 cell without vector treated with 1 μM puromycin (InvivoGen, San Diego, CA) were used as a negative control for this assay, with background set at 0% signal intensity. HEK293 cell with APOL1 G0 vector cells were set as 100% signal intensity.

### Cell viability

Cell viability assays were performed using ATP assays. To quantitate ATP generated by metabolically active cells, we used a CellTiter-Glo luminescent cell viability assay (Promega) per the manufacturer’s instructions. Cells were cultured in sterile 96-well plates in the presence of 10^3^ U per ml IFNα (Abcam) for 72 h, and then 100 μL of CellTiter-Glo reagent was added to lyse the cells. After a 10 min incubation at room temperature, luminescence was detected using a luminometer with an integration time of 1 s per well. The luminescence signals for cells were normalized by the cell count.

### Cell proliferation speed assay

Cells were seeded at 5.0 × 10^3^ cells per 150 µL culture medium into each well of 96-well plate and PKR inhibitor C16 was added at indicated concentration. After 0, 24, 48, 50 µL, cell number was measured by Cell Counting Kit-8 (Sigma-Aldrich).

### Oxygen consumption rates in cultured human podocytes

The XF24 extracellular flux analyzer (Seahorse Bioscience, Billerica, MA) was used to evaluate cellular oxygen consumption rates (OCR) according to the company’s directions. Briefly, cultured human podocytes were seeded in XF24 well plates purchased from Seahorse Bioscience, at a density of 2.0 × 10^4^ cells per well (surface area 0.33 cm^2^) in 100 μL culture medium and were incubated overnight at 37 °C. On the following day, the cells were treated with IFNα (1 × 10^3^ U per ml) with/without PKR inhibitor C16 (100 nM) for 72 h, followed by 24 h treatment on the XF24 well plates. Before OCR measurements, the experimental XF24 plate containing the cells was washed with bicarbonate-free DMEM assay medium (Seahorse Bioscience) containing 25 mM glucose and 1 mM sodium pyruvate, and the cells were preincubated for 1 h at 37 °C without a CO_2_ supply in 625 μL assay medium. Before OCR measurements, the XF24 was calibrated using a calibration cartridge according to the company’s directions. After the calibration, baseline OCR measurements were conducted in the test plate for 4 min.

### RNA-immunoprecipitation (RNA-IP)

Stable HEK293 cells bearing the APOL1-G0, G1, or G2 variant were mock-treated with the following: (a) 10^3^ U per ml IFNα for 24–72 h and 100 μM palmitic acid (Sigma) for 2 h, or (b) 100 mM 103 U per ml IFNα for 24–72 h and calyculin A for 30 min. Palmitic acid binds PKR directly and inhibits the dsRNA binding to PKR^44^ and therefore palmitic acid was used as negative control. Cells were irradiated with UV light at 200 mJ per cm^2^. Ten percent of each cell lysate was saved to serve as an input sample for RT-qPCR. Immunoprecipitation was performed using RNA-Binding protein immunoprecipitation kit (EMD Millipore) based on the previous report using PKR^45^. Phospho-PKR antibody (Santa Cruz Biotechnology, sc-101784) or control rabbit IgG was used for RNA-IP. RNA was extracted with TRIzol, followed by DNase treatment and ethanol precipitation. cDNAs were generated using random hexamer primers and SuperScript Reverse transcriptase II. Samples were analyzed by PCR (RT) or qRT-PCR using Power SYBR Green PCR master mix (ThermoFisher). Enrichment was calculated as a ratio (IP sample per input sample). Primer pairs are listed in Supplementary Table 1. No SYBR signal was detected in the negative controls, mock-treated cells with phospho-PKR antibody IP and interferon-α + calyculin A-treated cells with control rabbit IgG IP

### PKR protein expression and purification

Recombinant PKR was purified as previously reported^46^. PKR pPET-PKR/PPase (Addgene #42934) was transformed into BL21(DE3) Rosetta cells (Novagen). Cells were grown in LB medium at 37 °C until A_600 nm_ ~ 0.7 and protein expression was induced with 1 mM IPTG for 3 h at ~20 °C. For PKR purification, cells were resuspended in buffer A (20 mM HEPES (pH 7.5), 50 mM NaCl, 0.1 mM EDTA, 10 mM β-mercaptoethanol, 10% glycerol) supplemented with protease inhibitor cocktail (Sigma). Cells were lysed by incubation with 5 mg per ml of lysozyme for 30 min followed by sonication for 3 min. Lysate was centrifuged for 20 min at 20,000*g*. The supernatant was applied to a heparin Sepharose column (Amersham, Biosciences, Piscataway, NJ) equilibrated in buffer A and PKR was eluted using a NaCl gradient. The peak fractions were diluted twofold with buffer A and applied to a poly(I:C) agarose column (Amersham) equilibrated in buffer A. PKR was eluted at 1.1 M NaCl, concentrated to ~10 mg per ml and stored at −80 °C.

### PKR activation assay

PKR activation assays were performed as previously reported^47^. PKR was dephosphorylated by λ-protein phosphatase for 1 h at 30 °C and then inhibited with 2 mM sodium orthovanadate. PKR (4 μM) was incubated with long RNA (NM_001136540.1, 289–1453: 0.75 μM: 269 ng per ml)/short RNA (NM_001136540.1, 289–559/560–860/861–1179/1180–1453: 0.1 μM: 8.46 ng per ml)/poly (I:C) 20 μg per ml, 20 mM HEPES (pH 7.5), 4 mM MgCl_2_, 50 mM KCl, 1.5 mM DTT, and 100 μM ATP (Ambion). Reaction mixtures were incubated at 30 °C for the indicated times and quenched with 1× SDS loading buffer. Phosphorylated PKRs were detected with phospho-PKR antibody (Santa Cruz Biotechnology, sc-101784).

### RNA preparation for gel migration experiments and SHAPE

Truncated APOL1 RNAs with various 3′ structure cassettes were prepared by in vitro transcription using the MegaShortScript kit (ThermoFisher) according to manufacturers’ recommendations. Transcription templates were generated in two semi-nested PCR reactions from plasmids containing APOL1 alleles (G0, G1, G2) or mutant variants thereof (mG0, mG1, mG2). For the first reaction, forward and reverse primers were used to add a 5′ T7 promoter and a 45 nt 3′ SC, respectively, to the truncated *APOL1* sequences (Supplementary Table 1). The latter element is introduced to provide a reverse transcription hybridization site for SHAPE. A fraction of each reaction was re-amplified using the original forward primer and a shorter reverse primer to render the final transcription templates more homogeneous. Transcription reactions were treated with Turbo DNase I for 5 min at 37 °C, incubated at 85 °C for 2 min and fractionated over a denaturing gel (5% polyacrylamide—19:1, 1× TBE, 7 M urea) at constant temperature (45 °C, 30 W max). The desired RNA products were detected by UV shadowing, excised from the gel, electroeluted at 200 V for 2 h at 4 °C, ethanol precipitated and stored at −20 °C in 10 mM Tris, pH 7.0 prior to use. Stock RNA solutions were quantified by spectrophotometry.

### Gel migration assay

Each truncated APOL1 RNA was diluted to 1 μM in 20 μL RNA renaturation buffer (RB; 10 mM Tris (pH 8.0), 100 mM KCl, 0.1 mM EDTA, 5% glycerol (w per v)), denatured by heating to 85 °C for 2 min and renatured by slow cooling (0.1 °C per s) to 25 °C. MgCl_2_ was then added to a final concentration of 0, 1, or 3 mM and mixtures were incubated an additional 30 min at 37 °C to promote the formation of Mg-dependent and/or other tertiary RNA interactions. RNA conformers were stabilized by flash cooling to 4 °C, after which they were fractionated over a 5% non-denaturing polyacrylamide gel (5% acrylamide, 19:1) for 16–18 h at 4 °C. Both gel and running buffer contained 1× TBE and 1 mM MgCl_2_, and gels were pre-run for ~1 h prior to loading. Gels were exposed to SybrGreen dye (ThermoFisher) in accordance with the manufacturer’s instructions and the migration positions of RNA conformers detected using a Typhoon Trio+ variable mode imager (GE Healthcare).

### SHAPE and generation of RNA secondary structural models

SHAPE experimental methods have been described previously^48,49^. Briefly, the truncated APOL1 RNA with a SC RNAs were folded as in the gel migration assay except that the mixture volume was 150 μL, the final MgCl_2_ concentration was 1 mM and glycerol was excluded. RNA solutions were divided into control (1M7−) and experimental (1M7+) aliquots (72 μL each), and 8 μL DMSO or 8 μL 30 mM 1M7 in DMSO was added, respectively. Modification reactions were incubated at 37 °C for 5 min, cooled to 4 °C, ethanol precipitated and re-suspended in 10 μL nuclease-free water. Treated RNAs were reverse transcribed from differently labeled primers complementary to the 3′ structure cassette (1M7− primer, Cy5.5; 1M7+ primer, Cy5) and hydrolyzed by alkali treatment. The resultant cDNA libraries were precipitated with ethanol and re-dissolved in Sample Loading Solution (Genome Lab), then pooled with ddA and ddG sequencing ladders labeled with WellRED D2 (Beckman Coulter) and IRDye 800RS (LI-COR), respectively. Combined samples were fractionated by capillary electrophoresis (CE) using a Beckman Coulter CEQ 8000 Genetic Analyzer. For each RNA, reactivity profiles were generated from CE electropherograms using SHAPEfinder software^50^ and then inputted into RNAstructure software version 5.7^51,52^ together with the RNA sequences to generate secondary structural models. Default slope (1.8 kcal per mole) and intercept (−0.6 kcal per mol) parameters were used to transform reactivity values into the pseudo-energy constraints that modulate the RNA folding algorithm. RNAstructure automatically generates multiple structural models that best match the SHAPE-derived reactivity profiles and ranks them by Gibbs free energy. The lowest-energy structural models produced in this manner are illustrated in Fig. 3a, b and Supplementary Fig. 4.

### In vitro knock down

We conducted knock down experiments using conditionally immortalized human cell lines established from human urine^42^. We transfected these cells with either Stealth RNAi Negative Control Med GC (ThermoFisher) or with predesigned stealth siRNA against APOL1 (HSS112492, ThermoFisher) using Lipofectamine RNAiMAX (ThermoFisher). BLOCK-iT™ Alexa Fluor Red Fluorescent Oligo was co-transfected and used for transfection control. Knock down efficiencies were 52.4–75.4% (RNA levels) or 31.1% (protein levels).

### Mice

We used human *APOL1* gene locus transgenic mice (BAC-APOL1 mice), generated using a bacterial artificial chromosome which contains the locus for APLOL-G0, or APOL1-G1, or APOL1-G2. A ~47 kb human DNA, encompassing only the *APOL1 gene* with 5′ and 3′ flanking regions (including exons 1 and 2 of *APOL2* and 3′ region including exons 39–41 of part of *MYH9 gene*), was isolated and subcloned from human BAC clone (ENST00000397278, which corresponds to NM_003661). Individual G0, G1, and G2 BAC subclones were injected into 129SvJ/B6N F1 embryos and the founders were subsequently backcrossed into 129SvJ. The subclone was sequenced to ensure that it was as reference genome sequence from NCBI and Ensembl. The only differences were polymorphisms in the intronic regions. In the conditional transgenic mice experiments, we used podocyte-specific APOL1 truncated RNA transgenic mice of FVB background. We produced these mice by means of a conditional transgenic system using nephrin (*NPHS1*)-promoter-driven truncated RNA sequence (NM_001136540, 1031–1453). We generated two strains: NPHS1-APOL1-G0-delta-RNA with rs73885319 A and rs60910145 G and NPHS1-APOL1-G1-delta-RNA with rs73885319 G and rs60910145 G.

### Isolation of glomeruli

BAC/APOL1 mice (between 8 and 12 weeks old) were administered IFNγ (10^6^ U per kg body weight IP) daily for 3 days before tissue sampling. The glomerular isolation protocol has been reported in detail^53^. Briefly, mice were anesthetized by an intraperitoneal injection of Avertin (2,2,2-tribromoethyl and tertiary amyl alcohol; 17 μL per g) and perfused with 8 × 10^7^ Dynabeads (M450 tosylactivated: Dynal #140.04) diluted in 20 ml of phosphate-buffered saline through the heart. Kidneys were digested with collagenase A and DNase I at 37 °C for 10 min. The collagenase-digested tissue was gently pressed through a 100-μm cell strainer and washed with HBSS. The samples were washed and resuspended using a magnetic stand. Glomeruli were collected and incubated in RPMI1640 supplemented with 10% FBS, ITS, 1% penicillin/streptomycin (100 iU per ml; 100 μg per ml) and 100 mM calyculin A (LC Laboratories) for 30 min and used for Western blot analysis.

### Immunohistochemistry (mouse specimens)

BAC/APOL1 mice (between 8 and 12 weeks old) were administered IFNγ (10^6^ U per kg body weight IP) daily for 3 days. Mice are perfused with 100 mM calyculin A contained PBS before tissue sampling. Then kidneys were cut into small pieces and incubated in RPMI1640 supplemented with 10% FBS, ITS, and 100 mM calyculin A for 30 min. Then tissues were immediately fixed with 10% buffered formalin. BAC/APOL1 Mice (between 8 and 12 weeks old) were administered IFNγ (10^6^ U per kg body weight IP) daily for 3 days. NPHS1-APOL1-ΔRNA mice were administered IFNγ (10^6^ U per kg body weight IP) daily for 3 days before tissue sampling. We used paraffin fixed 4–5 μm tissue sections. The sections were deparaffinized/rehydrated, antigen retrieval performed by heating in citrate-buffered medium for 5 min in a microwave. Tissues were blocked by 1% BSA and 0.1% saponin. Sections were incubated with primary antibodies including the following: APOL1 (Sigma Aldrich HPA018885), phospho-PKR (Santa Cruz Biotechnology sc-16565), phospho-PKR (Santa Cruz Biotechnology Inc. sc-101784), WT1 (Santa Cruz Biotechnology sc-192), and podocalyxin (R&D Systems AF1556). For immunofluorescence, we used Alexa Fluor secondary antibodies (ThermoFisher) and visualized using confocal microscopy. For ABC stain, biotin conjugated secondary antibody and Avidin-HRP (Vector) were used. A DAB kit (Vector) was used for visualization. In mouse experiments, mice (between 8 and 12 weeks old) were treated with IFNγ (10^6^ U per kg body weight IP), injected for 3 days before tissue sampling. For signal quantification, phospho-PKR signal of podocyte was detected by counter stain for WT1 in mouse experiments.

### Immunohistochemistry (human specimens)

This study was approved in advance by IRB in University of Maryland and of the NIDDK, NIH. Basic characteristics of cases are listed in Supplementary Fig. 5b. Staining was performed using 5 μm-thick formalin-fixed, paraffin-embedded tissue sections. Following deparaffinization, heat-induced antigen retrieval was performed for 20 min in a buffer of pH 6.0 using a pressure cooker (Pascal, Agilent Technologies Dako). Sections were incubated with primary antibodies including the following: phospho-PKR (Santa Cruz Biotechnology sc-16565) and WT-1 (Santa Cruz Biotechnology sc-192). We used Alexa Fluor secondary antibodies (ThermoFisher) and visualized using confocal microscopy.

### Immunohistochemistry and live cell imaging

Cells were incubated with 200 nM of Mito Tracker Green (Invitrogen) and 200 nM of TMRE (Invitrogen) for 20 min prior to signal detection, after 20 μM FCCP treatment and visualized using confocal microscopy.

### Signal quantification of microscopic images

For human tissue, we counted glomeruli without global sclerosis (6 non-risk genotype cases: 5, 6, 7, 10, 5, and 9 glomeruli for each case, respectively; 6 risk-genotype cases: 13, 6, 4, 5, 10, and 5 glomeruli, respectively). For mouse and human tissues, laser power was adjusted so that maximal signal was not saturated. Image processing and colocalization analyses were performed using the Zen software (Carl Zeiss, Oberkochen, Germany)^54^. Weighted colocalization coefficient and mean intensity in WT-1 positive cells were calculated as previously reported^54^. For the mean intensity, we standardized the podocyte signal intensity using signals from WT1-negative intra-glomerular cells for each glomerulus. The values were relative score with signal intensity of APOL1-G0 as 100%. Quantifications were performed in a blinded manner.

### In situ hybridization

Chromogenic in situ detection was performed on tissue sections from the mouse formalin-fixed paraffin-embedded (FFPE) blocks using the RNAscope in situ hybridization (Advanced Cell Diagnostics, Biotechne, Minneapolis, MN). Briefly, 5 μm FFPE tissue sections were de-paraffinized, boiled with pretreatment reagent for 15 min, and then protease digested at 40 °C for 30 min, followed by hybridization for 2 h at 40 °C with probe-Hs-APOL1-01 (Catalog # 439871, Advanced Cell Diagnostics). In addition, Probe-Mm-PPIB (Catalog # 313911) and Probe-DapB (Catalog # 310043) were used for positive and negative control, respectively. Detection of specific probe binding sites was visualized with RNAScope 2.0 HD Reagent Kit (Brown) (Catalog # 310035).

### Mouse proteinuria model

All experiments were conducted in accordance with the National Institutes of Health Guide for the Care and Use of Laboratory Animals and were approved in advance by the NIDDK Animal Care and Use Committee (Animal study proposal (K097-KDB-14)). We used both male and female mice, aged 8–15 weeks. Mice in each experiment were matched for sex, age, and body weight. Randomizations were performed with regard to body weight. Sample sizes for experiments were determined without formal power calculations. Exclusion criteria were weight loss more than 20% during the experimental period. For proteinuria induction, mice (between 8 and 12 weeks old) were injected with bFGF and puromycin aminonucleoside, as previously described^30^. For NPHS1-APOL1-deltaRNA mice strain, puromycin aminonucleoside (Sigma-Aldrich) was injected subcutaneously at day 0 (200 mg per kg body weight), and bFGF (Kaken Pharmaceutical) was injected intravenously at days 0 and 2 (5 μg per animal), respectively. For PKR inhibitor experiments, PKR inhibitor C16 (10 μg per kg IP) daily from day 0 to day 10^55^. For BAC-APOL1 mice strain, interferon γ (Prospec) was injected at day −1 and day 1 (10^6^ U per kg body weight), puromycin aminonucleoside was injected subcutaneously at day 0 (300 mg per kg body weight), and bFGF (Kaken Pharmaceutical) was injected intravenously at days 0 and 2 (5 μg per animal), respectively. For PKR inhibitor experiments, PKR inhibitor C16 (10 μg per kg IP) daily from day 0 to day 10^55^.

### Urinary albumin and creatinine measurement

We determined the urinary albumin levels with ELISA using a murine microalbuminuria ELISA kit (Exocell). We measured the urine creatinine concentration with creatinine kit (Exocell). All measurements were performed in duplicate. We determined albuminuria as the ratio of urinary albumin to creatinine. All procedures were performed in accord with the manufacturers’ protocols. Investigators were not blinded to group allocation but were blinded when assessing outcome.

### Statistical analysis

Data from at least three individual experiments were analyzed and presented as dot plot and mean. Statistical analysis methods were written in each figure legends. We did not adjust for multiple comparisons. Values of *P* < 0.05 were considered statistically significant.

## Electronic supplementary material

Supplementary file

## Data Availability

Raw gel images are available in Supplementary Figure 13. The datasets generated or analyzed during the current study are available from the corresponding author on request.
