# Supplementary material for: APOL1 risk allele RNA contributes to renal toxicity by activating protein kinase R
Source: Commun Biol. 2018 Nov 7;1:188. doi: 10.1038/s42003-018-0188-2 (PMC6220249; doi:10.1038/s42003-018-0188-2)

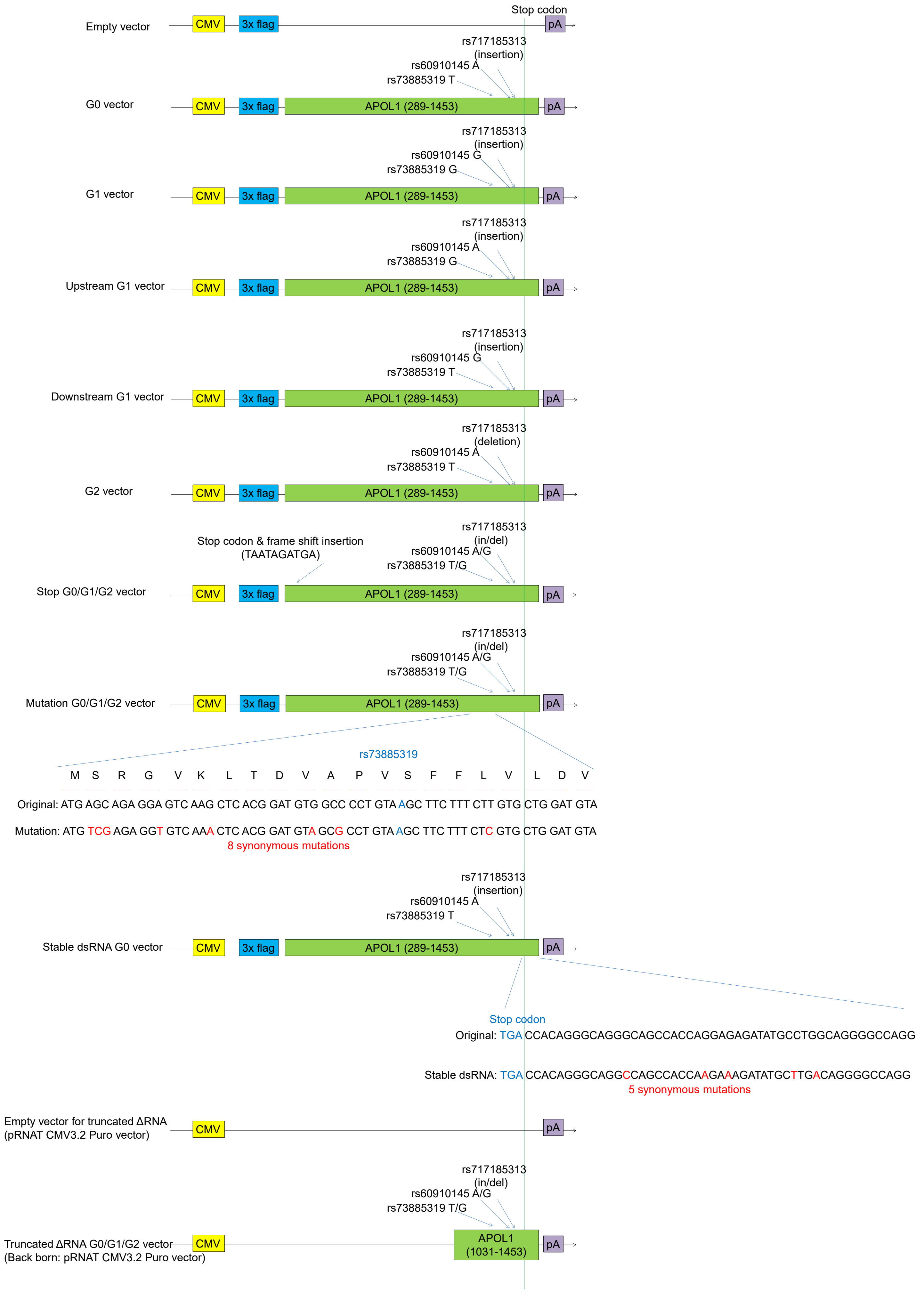

Supplementary Figure 1
Shown are the constructs used In the experiments described herein, with the location of the two renal risk variants, the two G1 mutations (referred to in the text as upstream and downstream respectively) and G2 six-base pair deletion.

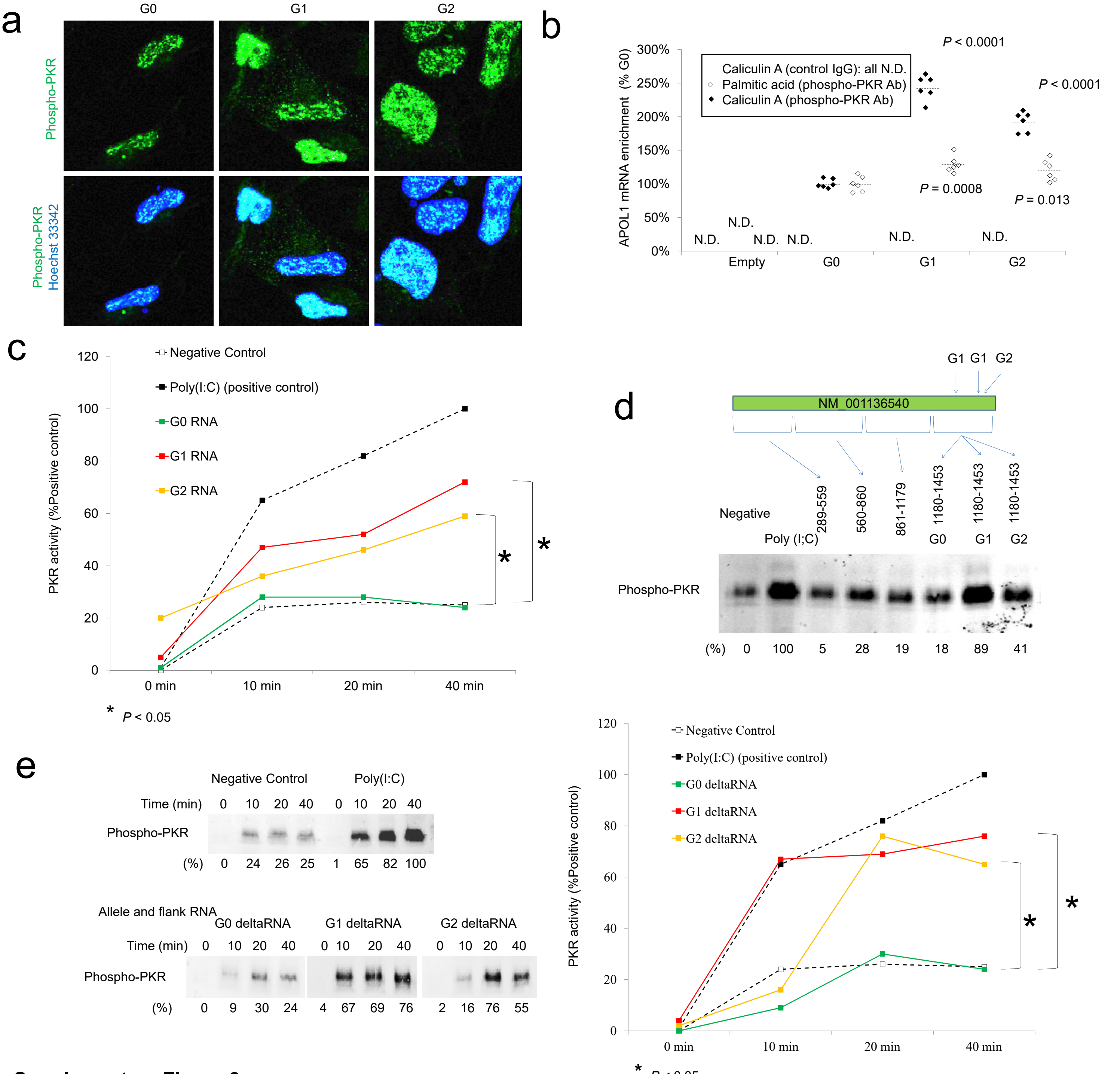

Supplementary Figure 2

(a) Stable HEK293 cell lines expressing APOL1. The G0 variant showed minimally increased phospho-PKR, indicating activation, while renal risk variants G1 and G2 manifested greatly increased phospho-PKR.

(b) RNA immunoprecipitation from stable HEK293FT cell lines expressing APOL1. The graph shows relative enrichment of APOL1 mRNA that was cross-linked to phospho-PKR in the G0 cells set to 100%. In the presence of global phosphatase inhibitor (caliculin A), G1 and G2 mRNAs were enriched compared with G0 mRNA [show these data on the left]. In cells exposed to caliculin A plus palmitic acid (an eIF2α pathway inducer direct inhibits PKR) , the enrichment in APOL1 RNA cross-linked to phospho-PKR was reduced.

(c) APOL1 G1 and G2 RNA promote PKR activation in a time-dependent manner. RNAs (NM_001136540.1, 298-1453) transcribed *in vitro* using T7 sequence were incubated with PKR and ATP for indicated times. Poly(I:C) served as the positive control for PKR phosphorylation. Vehicle was used for negative control.

(d) Truncated APOL1 RNAs (NM_001136540.1, 1180-1453) containing the G1 and G2 variants was sufficient to enhance PKR activation. RNAs transcribed *in vitro* using T7 sequence were incubated with PKR and ATP for 20 min. Poly(I:C) served as the positive control for PKR phosphorylation. Vehicle was used for negative control. Western blots identifying phospho-PKR are presented. The quantified intensity of phospho-PKR to each RNAs was added below the gel images.

(e) Truncated G1 and G2 RNA (NM_001136540.1, 1180-1453) promote increased PKR activation in a time dependent manner, compared to truncated G0 RNA. Synthetic RNAs (0.10 μM) were incubated with 4 μM PKR and ATP for the indicated times. Poly(I:C) served as the positive control for PKR phosphorylation. Vehicle was used as the negative control. Western blots identifying phospho-PKR are presented. The quantified intensity of phospho-PKR to each RNA was added below the gel images.

P values were calculated using a Student one-tailed t-test (b) or a Wilcoxon t-test (c & e). $P < 0.05$ is statistically significant.

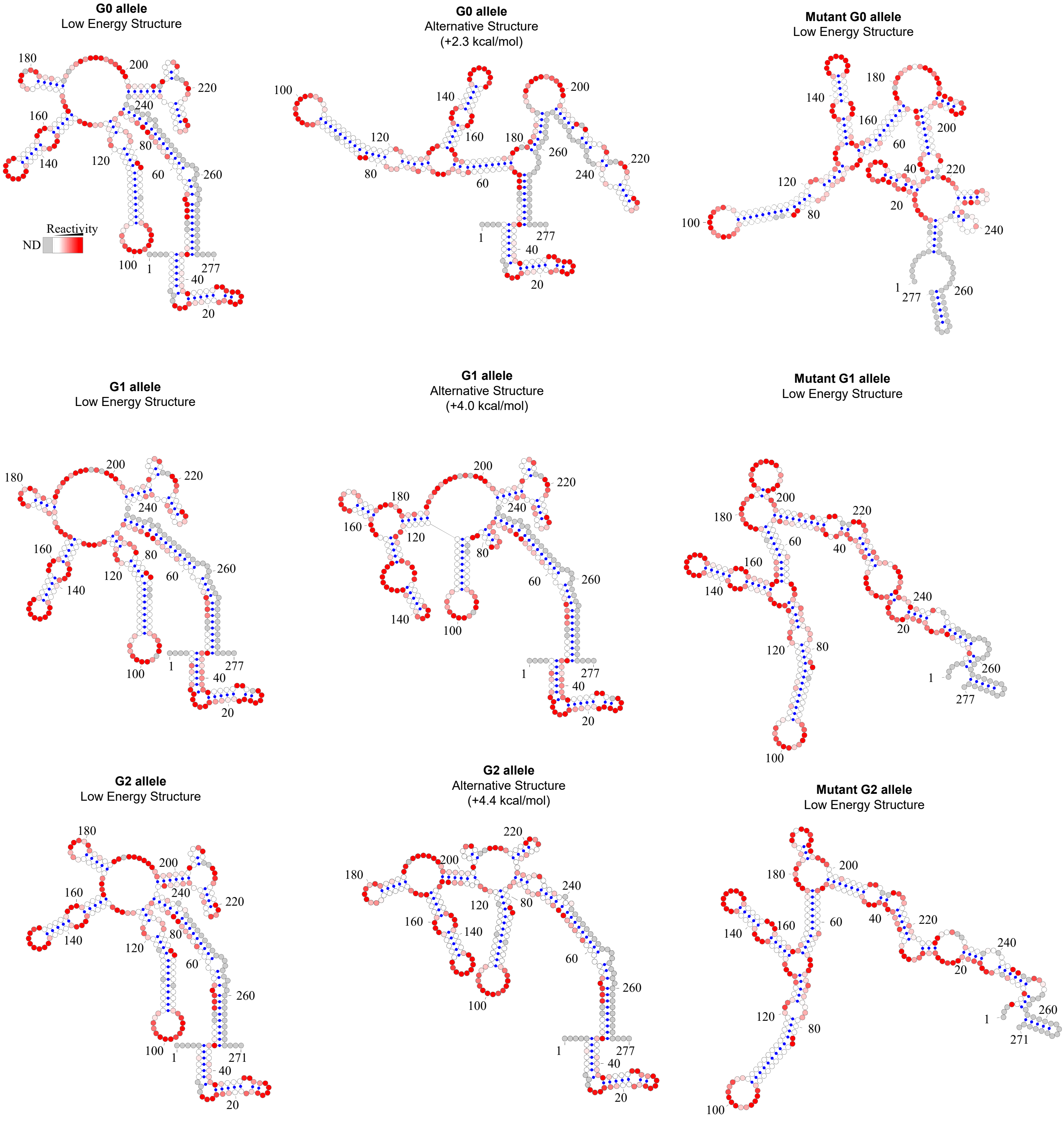

Supplementary Figure 3.
SHAPE reactivity maps for truncated APOL1 allelic variant and mutant RNAs. The three rows show SHAPE data for the G0, G1 and G2 allelic variants. Shown in each row are SHAPE-derived native APOL1 secondary structural models of lowest energy (left), second-lowest energy (center), and mutated APOL1 secondary structural models of lowest energy (right).

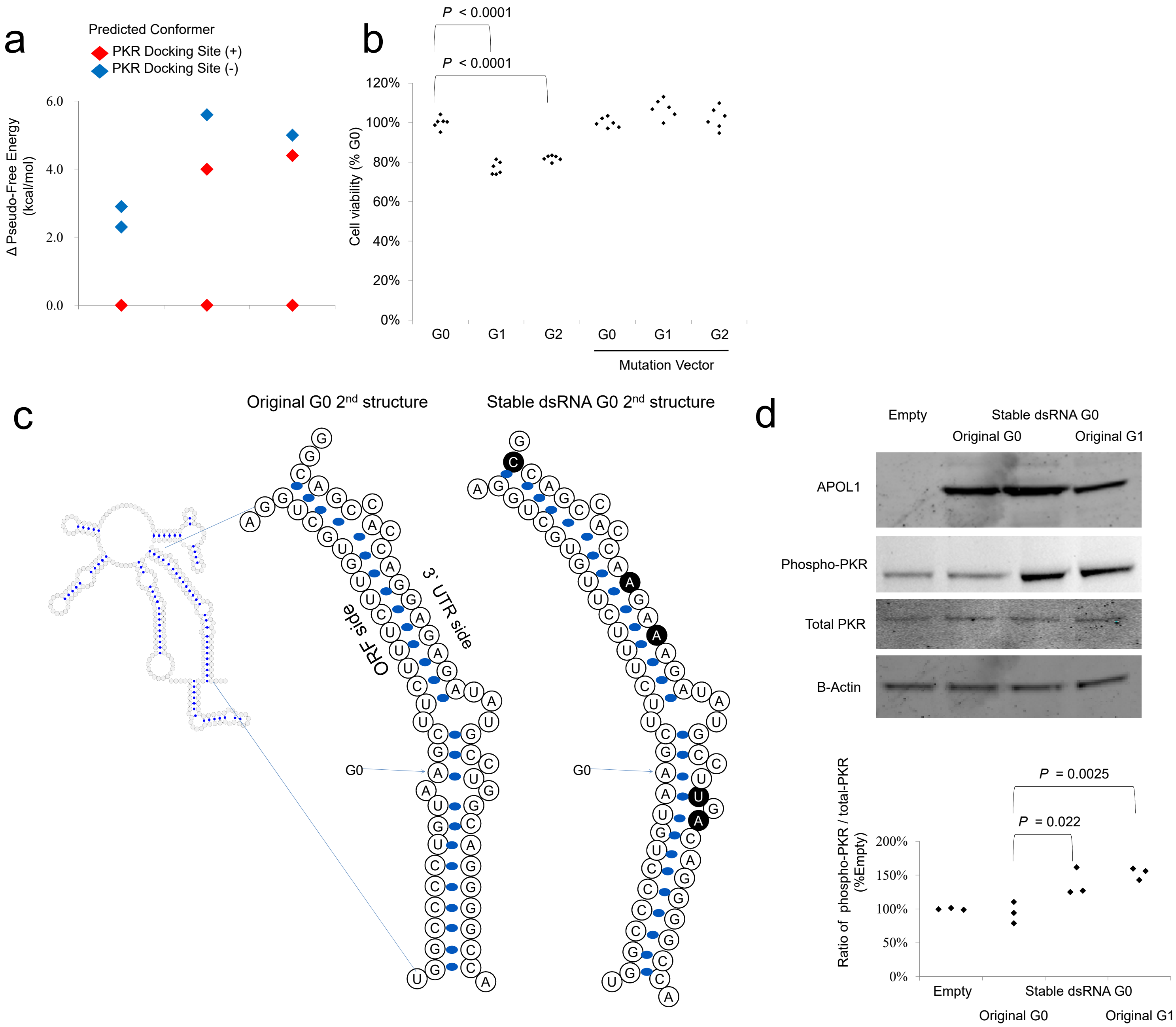

Supplementary Figure 4.

(a) Structural models for truncated APOL1 RNA variants were generated using RNAstructure software from SHAPE-derived reactivity profiles. The software converts RNA reactivity values into pseudo-free energy constraints that are then assimilated into the secondary structure prediction algorithm. In general, numerous structural models are generated for every data set and ranked by collective pseudo-free energy. Structures having relatively low pseudo-free energy values are considered more stable and likely to have a greater prevalence in structurally heterogeneous populations. Relative pseudo-free energies are given here for the three lowest energy conformers for each of the G0, G1 and G2 truncated APOL1 RNA variants. To allow for direct comparision of relative energies, the most stable predicted conformer in each of the respective groups is set to zero (0) kcal/mol. Whether or not a predicted conformer contains a PKR docking site is also indicated. The absolute pseudo-free energies determined for the lowest (1L) were G0 -175.6, G1 -159.3, G2 -164.1 kcal/mole, resplectly.

(b) Quantitation of cell viability of stable HEK293FT cell line expressing APOL1. The cells were lysed and incubated with CellTiter-Glo to measure the amount of ATP in the cells. Cell viability of G1 and G2 variants decreased cell viability and recovered with synonymous mutations.

(c) To make stable dsRNA form of APOL1 G0 RNA variant based on the lowest-energy secondary structural model, 5 nucleotides were changed (black ball) refer to complimentary sequence.

(d)Stable HEK293FT cell lines expressing APOL1 variants were harvested. Constructs expressing stable dsRNA type APOL1 G0 variant increased phospho-PKR as well as G1 variant.

All results are presented as ratio of controls (G0 or empty) was normalized to 100% and P values were calculated using a Student one-tailed t-test.

a

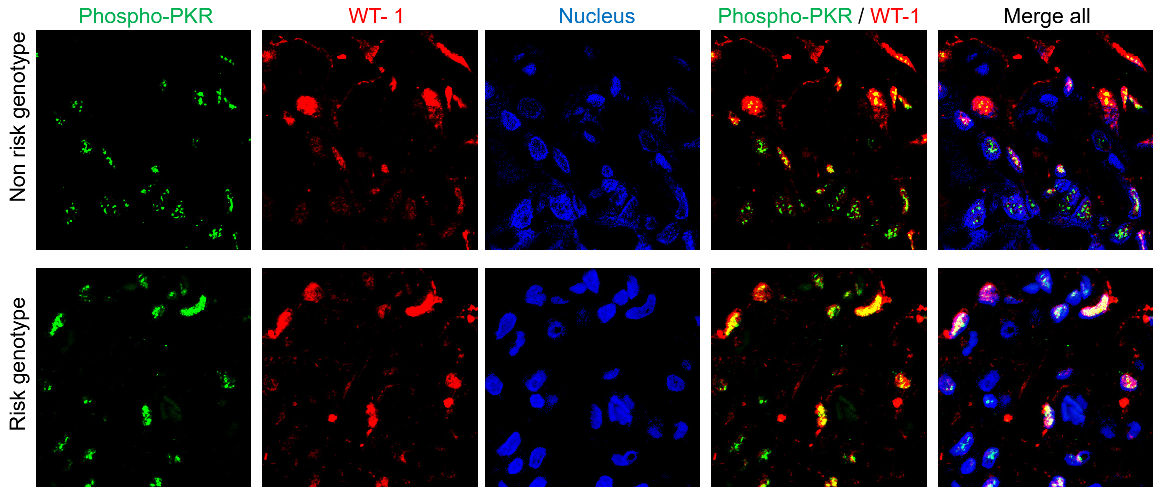

b

		Pathological Diagnosis	Race	Sex	Age at biopsy	Genotype
0 risk allele (Non risk genotype)	Case 1	FSGS	African American	M	29	G0/G0
	Case 2	FSGS	Caucasian	M	51	G0/G0
1 risk allele (Non risk genotype)	Case 1	FSGS	African American	M	11	G0/G1
	Case 2	FSGS	African American	M	40	G0/G2
	Case 3	FSGS	African American	F	28	G0/G2
	Case 4	FSGS	African American	M	18	G0/G1
2 risk alleles (Risk genotype)	Case 1	FSGS	Unknown	F	57	G1/G2
	Case 2	FSGS	African American	M	13	G1/G2
	Case 3	FSGS	African American	F	34	G1/G1
	Case 4	FSGS	African American	M	35	G1/G2
	Case 5	FSGS	African American	F	32	G1/G1
	Case 6	FSGS	African American	M	17	G1/G1

c

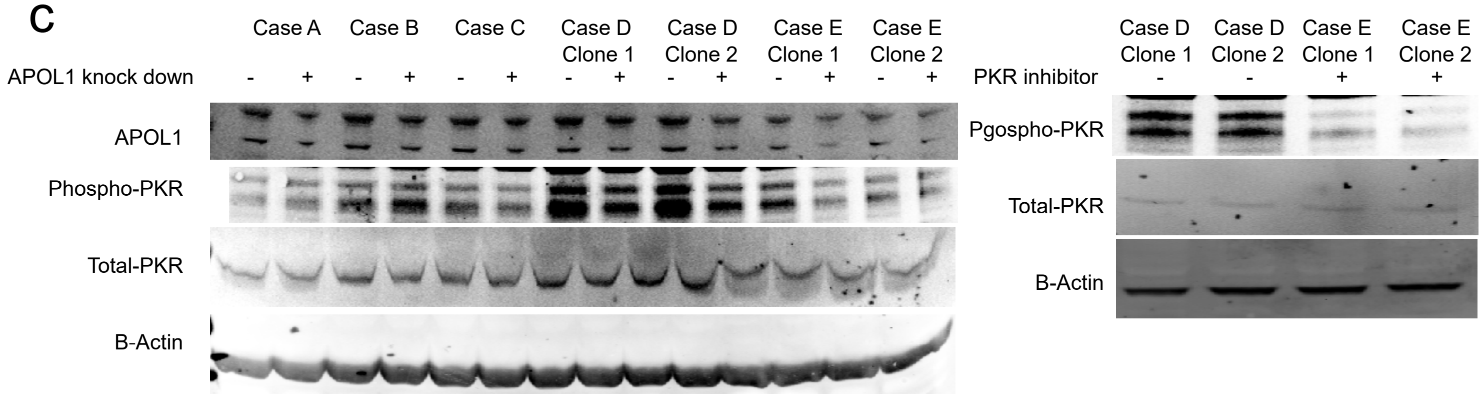

Supplementary Figure 5

(a) Immunofluorescence staining of human glomerulus was visualized using confocal microscopy. Phospho-PKR was visualized as green, APOL1 as red and nucleus as blue. Green signal overlapped with red and blue localized phospho-PKR signal to podocytes and green signal overlapped with only blue indicated phospho-PKR signal in other glomerular cells.

(b) Basic characteristics of each biopsy case.

(c) Left: Conditionally immortalized human podocytes, one cell line from each of three APOL1 G0/G0 FSGS patients and two cell lines from each of two APOL1 G1/G2 FSGS patients, were transfected with APOL1 siRNA or control siRNA. Phospho-PKR signal / Total-PKR was measured from blot. Knock down efficiencies were 52.4-75.4% (RNA levels) or 31.1% (protein levels). G1/G2 podocytes manifested increased PKR phosphorylation, which was diminished by APOL1 RNA knock-down. Right: Glomeruli were treated with phosphatase inhibitor with/without PKR inhibitor for 30 min and lyzed for analysis. The phospho-PKR bands reduced in the presence of PKR inhibitor

a

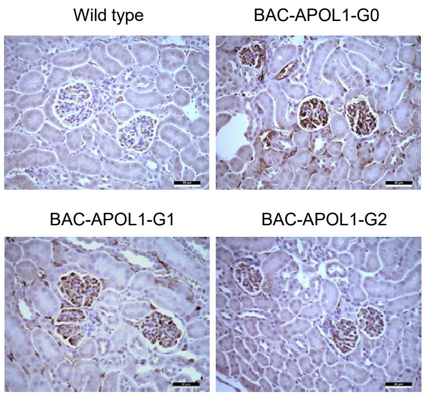

b

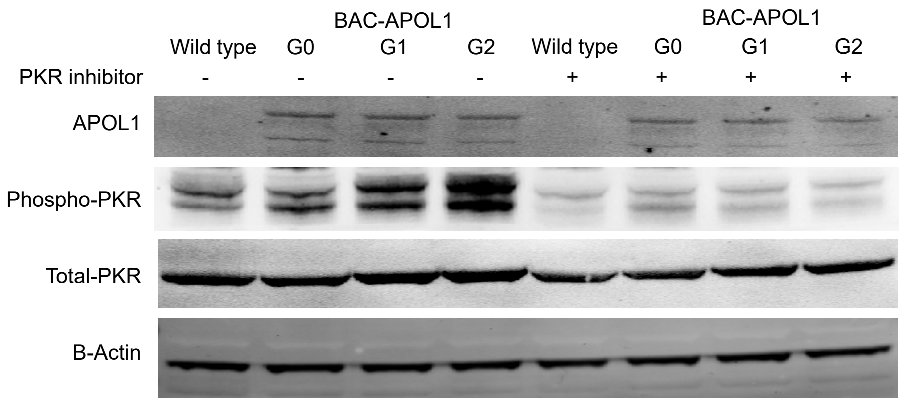

c

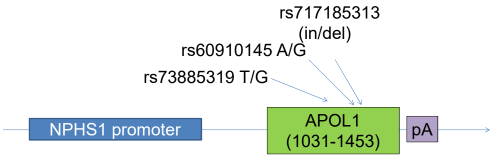

	rs738853	rs60910145	rs717185313
NPHS1-APOL1 –G0-deltaRNA	T	T	Insertion
NPHS1-APOL1 -G1-deltaRNA	G	G	Insertion

d

Cycle threshold (Ct) value	Wild	NPHS1-APOL1 –G0-deltaRNA	NPHS1-APOL1 –G1-deltaRNA
APOL1-RNA RT(+)	33.549	23.109	23.078
	32.601	23.612	23.634
	33.287	24.251	24.217
APOL1-RNA RT(-) pooled	33.200	32.477	29.819
B-Actin	15.901	14.799	14.858
	15.980	15.218	15.367
	14.653	15.108	15.225

e

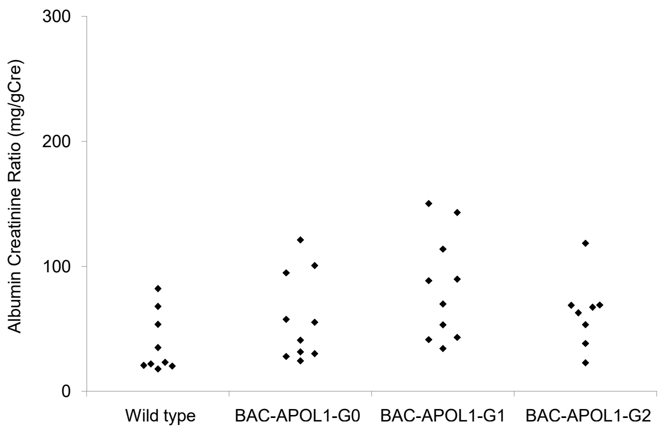

f

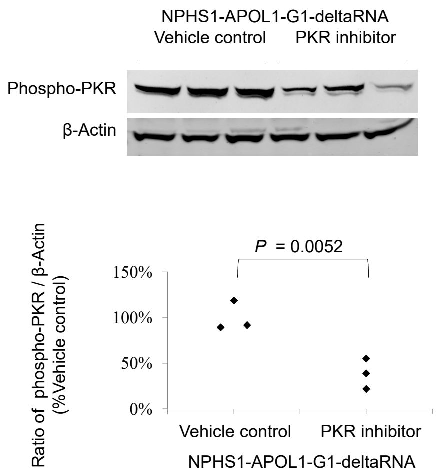

Supplementary Figure 6

(a)APOL1 expression in transgenic mice with human APOL1 gene locus. Sections of mice kidney cortex stained with antibody against APOL1, demonstrating glomerular expression in all three APOL1-expressing mouse lines but not in wild-type mice.

(b) Glomeruli were isolated from BAC-APOL1 transgenic mice using magnetic particle (upper panel: arrows indicated glomeruli, small dots are magnet beads). Glomeruli were treated with phosphatase inhibitor with/without PKR inhibitor for 30 min and lysed for analysis. Phospho-PKR was increased in G1 and G2 glomeruli. Phospho-PKR signal reduced in the presence of PKR inhibitor

(c) NPHS1-APOL1-deltaRNA mouse. Transgene schema is shown.

(d) Quantitative-PCR detected APOL1-deltaRNA mRNA in renal cortex from transgenic mice demonstrates transgene expression in G0 and G1 mice.

(e) Urine albumin/creatinine ratio (mg/g) was measured in BAC-APOL1 transgenic mice. Risk variant transgenic mice did not manifest albuminuria, even at 8-12 weeks of age.

(f) NPHS1-APOL1-G1-delta-RNA transgenic mice received a PKR inhibitor or vehicle. Western blot of kidney cortex lysates demonstrated reduced phospho-PKR in NPHS-APOL1-G1-deltaRNA mice with the PKR inhibitor compared to vehicle control.

P values were calculated using a Student one-tailed t-test.

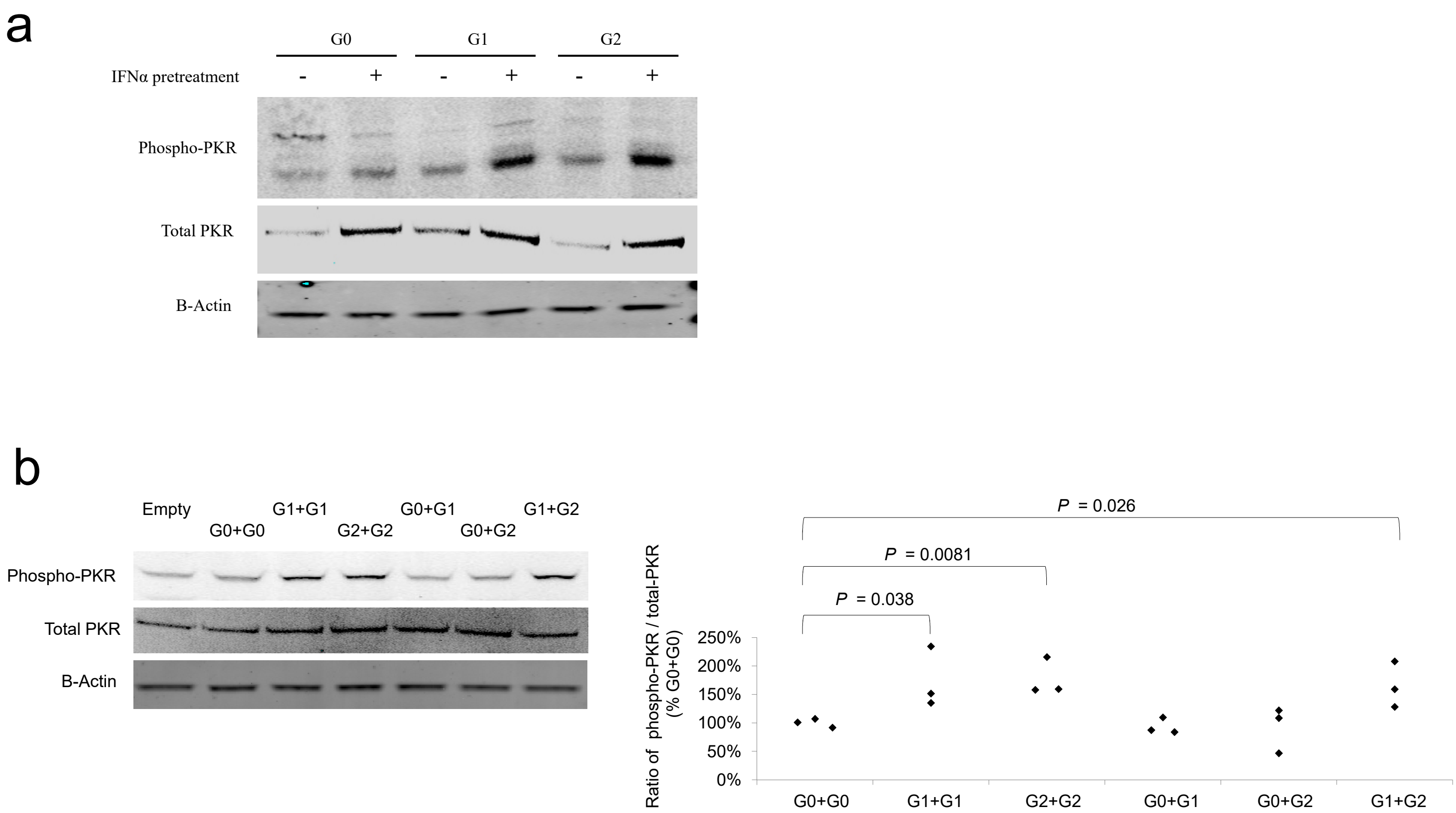

Supplementary Figure 7

(a) HEK293FT cells over-expressing APOL1 were harvested after pretreatment of interferon α for 72 hours. Induced PKR expression increased phospho-PKR in the G1 and G2 variants, while the G0 variant showed minimally increased phospho-PKR.

(b) Transient transfection of HEK293 cells with each APOL1 variant. The non risk genotype model (G0/G0, G0/G1 and G0/G2) transfection cells showed minimally increased phospho-PKR, while the risk genotype model (G1/G1, G2/G2 and G1/G2) cells manifested greatly increased phospho-PKR.

Results are presented as ratio of controls (G0/G0) was normalized to 100% and P values were calculated using a Student one-tailed t-test.

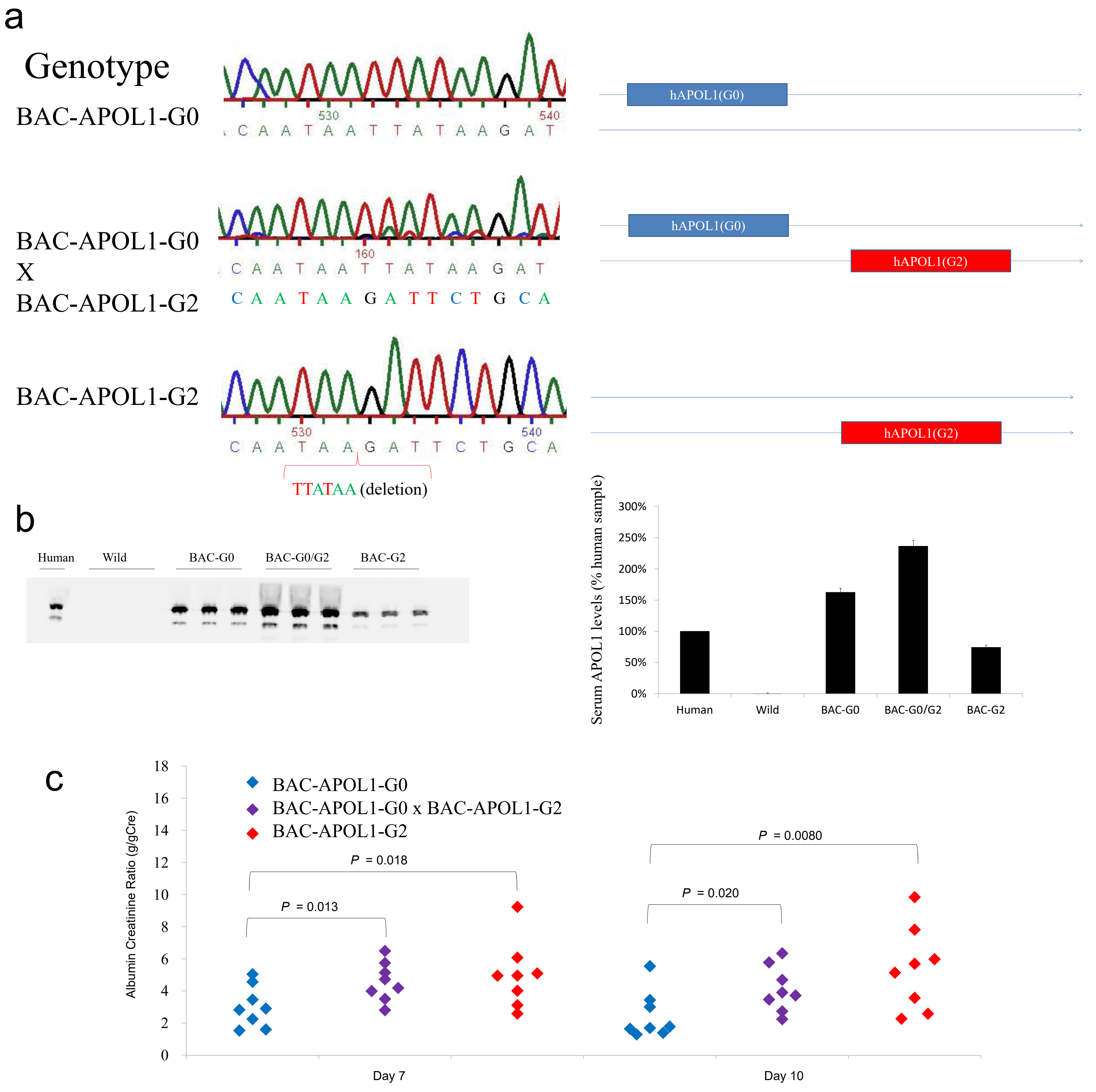

Supplementary Figure 8

(a) The sequence results from genomic DNA of APOL1 allele region, to confirm the genotypes of the single and double transgenic.

(b) Western blot analysis of APOL1 from mouse and human serum (positive control). Each well contains an equal volume of human or mouse serum (1 μl). In the bar graph each group had 3 mice. Data are mean ± SD.

(c) BAC-APOL1 transgenic mice manifested more proteinuria following podocyte injury, assessed as albumin/creatinine ratio (g/gCre), with higher levels in BAC-APOL1-G2 transgenic mice compared to BAC-APOL1-G0 transgenic mice. Urine protein was measured on days 7 and 10 after initiation of interferone γ, puromycin aminonucleoside and basic FGF exposure, which together induce podocyte injury. Each value represents data from one mouse. BAC-APOL1-G0 and G2 values are the same as main Figure 4d. Results are presented as means ± s.d. P values were calculated using a Student one-tailed t-test.

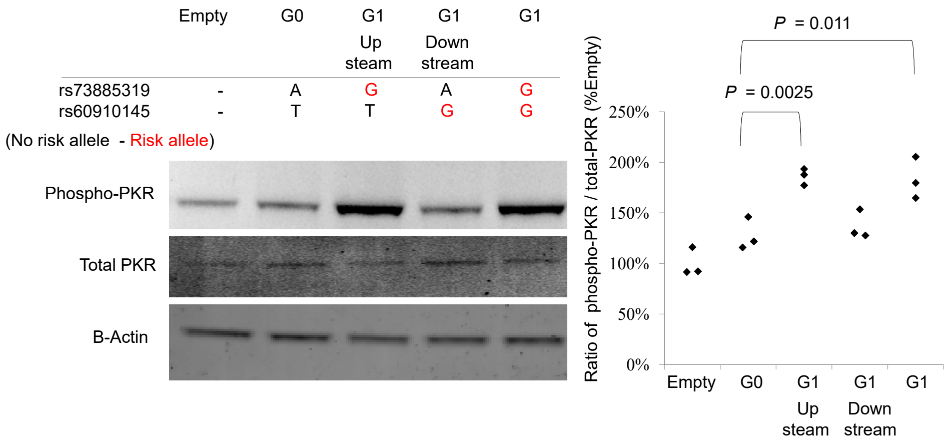

Supplementary Figure 9

Transiently-transfected HEK293 cell lines expressing APOL1. The G0 and downstream G1 rs60910145 G variant showed minimally increased phospho-PKR, while the upstream G1 rs73885319 G variant manifested greatly increased phospho-PKR comparable to the G1 allele containing both upstream and downstream variants. All results are presented as ratio of controls (empty) was normalized to 100% and P values were calculated using a Student one-tailed t-test.

a

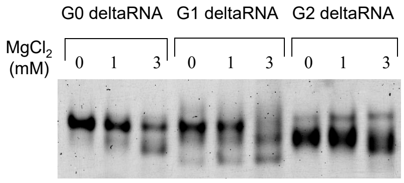

Supplementary Figure 10.

Non-denaturing PAGE analysis of partial RNA sequence. SybrGreen stain of the truncated APOL1 RNA folded in the absence or presence of 1 or 3 mM MgCl2 and run on a 5% native gel. Heterogeneity of APOL1 RNA gel mobility is apparent, increasing in the presence of higher Mg concentrations.

a

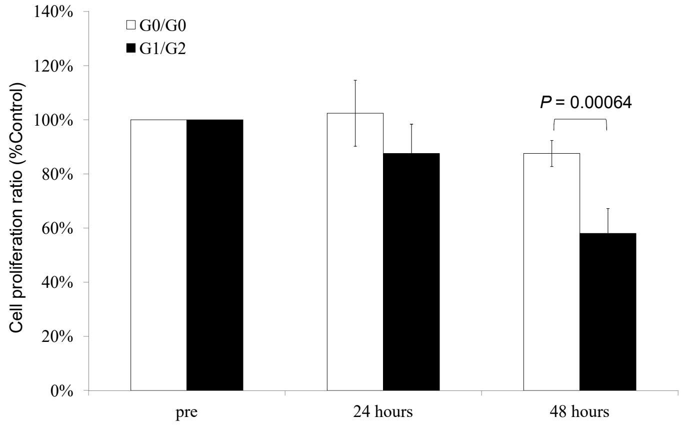

b

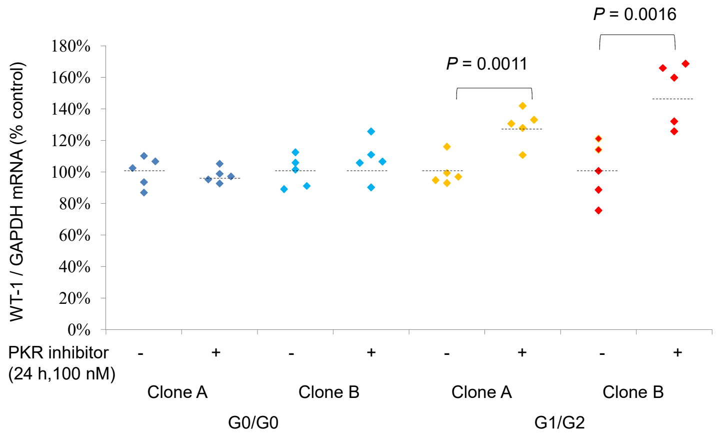

Supplementary Figure 11

(a) Effect of PKR inhibitor (C16, 100 nM) on the cell proliferation rate of undifferentiated human podocytes, expressed as per cent values obtained with vehicle (N = 4).

(b) In G1/G2 cultured human podocytes, PKR inhibitor restored the podocyte marker (WT-1) compared to vehicle treatment, as measured by qRT-PCR, while the PKR inhibitor had no effect on WT-1 expression. Results are presented as ratio of controls (vehicle) was normalized to 100% and P values were calculated using a Student one-tailed t-test.

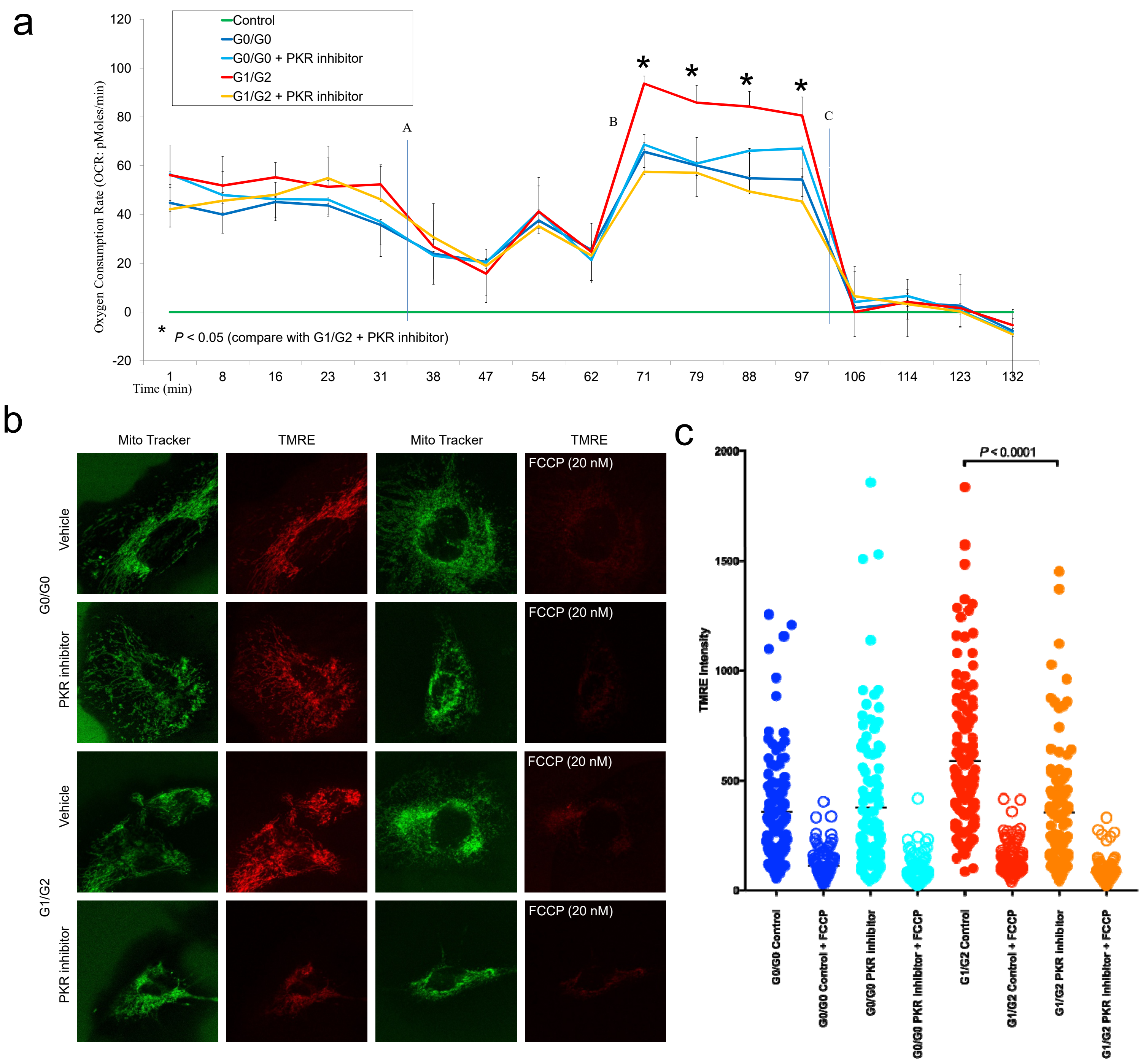

Supplementary Figure 12

(a) The PKR inhibitor decreased mitochondrial respiration of G1/G2 cultured human podocytes. Cells were seeded and reached a final density of 20,000 cells/well. Effects of the PKR inhibitor were compared using a Seahorse XF-24 extracellular flux analyzer to measure oxygen consumption rate (OCR) in the presence of metabolic inhibitors and uncoupling agents. The first injection (shown as vertical line A), oligomycin, an inhibitor of ATP synthesis. This agent distinguishes the fraction of OCR attributed to ATP synthesis by blocking the oxygen consumption required to overcome proton leakage across the inner mitochondrial membrane; it also provides the basal respiration rate by blocking non-mitochondrial respiration. The second injection (shown as vertical line B) was an uncoupling agent (FCCP) that disrupts ATP synthesis. This agent is used to calculate the spare respiration capacity of cells, defined as the quantitative difference between maximal and basal respiration rates. The third injection (shown as vertical line C) was a combination of a complex I inhibitor (rotenone) and a complex III inhibitor (antimycin A); this combination blocks mitochondrial respiration and enables calculation of mitochondrial and non-mitochondrial cellular respiration. Data were expressed as mean$\pm$SD at each time point. P values were calculated using a Student one-tailed t-test. $P < 0.05$ is statistically significant. (n = 3)

(b) (c) PKR inhibitor decreased mitochondrial membrane potential of G1/G2 cultured human podocytes. cultured human podocytes were incubated for48 hours with a final concentration of 1000 U/ml interferone alfa with/without 1 μM PKR inhibitor. Cells were incubated for 20 min with a final concentration of 200 nM Mito tracker and 200 nM TMRE (tetramethylrhodamine ethyl ester, a live cell fluorescence marker of mitochondrial membrane potential). P values were calculated using a Student one-tailed t-test.

Supplementary Table 1.

Primer pair for quantitative real-time PCR		
Target Gene	Forward	Reverse
Interferon α	GAC TCC ATC TTG GCT GTG A	TGA TTT CTG CTC TGA CAA CCT
Interferon β	TGG GAG GAT TCT GCA TTA CC	CAG CAT CTG CTG GTT GAA GA
β-Actin	CGC ACC ACT GGC ATT GTC	TTC TCC TTG ATG TCA CGC AC
APOL1	CAG CCT TGT ACT CTT GGA ACC	GCT TTT GAT GAC CAG GTC GTG
GAPDH	GAA ATC CCA TCA CCA TCT TCC AGG	GAG CCC CAG CCT TCT CCA TG
WT-1	GAG AGC GAT AAC CAC ACA ACG	AGA TGC CGA CCG TAC AAG AG

Primer pair for RT-PCR		
Target Gene	Forward	Reverse
β-Actin	CGC ACC ACT GGC ATT GTC	TTC TCC TTG ATG TCA CGC AC
APOL1	CAA TGT GGT GTC TGG CTC TCT C	AAT GCC TCG TGT GAG TTG GTA AG
Truncated APOL1 RNA	ATC CTC ACA CGA GGC ATT G	TAG AAG GCA CAG TCG AGG

Primer pair for quantitative real-time PCR in RNA-IP		
Target Gene	Forward	Reverse
APOL1	CAG CCT TGT ACT CTT GGA ACC	GCT TTT GAT GAC CAG GTC GTG

Primers for SHAPE	
Foword primer for 1st reaction	GAC GTA ATA CGA CTC ACT ATA GGG AGC ATC CTG GAA ATG
Revsverse primer for 1st reaction	GAA CCG GAC CGA AGC CCG ATT TGG ATC CGG CGA ACC GGA TCG ACC TGC CCC TGC CAG GCA TAT CTC TCC TGG TGG CTG CCC TGC CCT GTG GTC ACA GTT CTT GGT CCG CC
Revsverse primer for re-amplification	GAA CCG GAC CGA AGC CCG ATT T

Supplementary Table 2.

Raw values of Figure 4c

Sample ID	Control siRNA			APOL1 siRNA		
Case A	0.83076	0.826204	0.870411	1.000000	1.000000	1.000000
Case B	0.916006	0.80225	0.949359	1.000000	1.000000	1.000000
Case C	1.200943	1.060484	0.928268	1.000000	1.000000	1.000000
Case D-Clone 1	1.399687	1.689202	1.304097	1.000000	1.000000	1.000000
Case D-Clone 2	1.395837	1.250508	1.173099	1.000000	1.000000	1.000000
Case E-Clone 1	1.349704	1.651641	1.573226	1.000000	1.000000	1.000000
Case E-Clone 2	1.669596	1.379549	1.433107	1.000000	1.000000	1.000000

Supplementary Table 3.

Raw values of Figure 4d

Sample ID	Control siRNA						APOL1 siRNA					
Case A	1.083235	0.785931	1.01537	1.409173	1.080818	0.980601	0.999194	1.121351	0.942485	0.812891	1.262845	0.861233
Case B	1.164605	1.055821	1.124832	1.173833	1.045776	0.686594	1.058434	1.071093	0.931602	1.141084	0.972682	0.825105
Case C	0.984491	1.112477	1.061535	1.162254	1.108498	0.97993	0.891437	0.898423	1.501189	0.990992	0.921032	0.796927
Case D-Clone 1	0.754687	0.978956	0.882466	0.822505	0.840287	0.872817	1.037815	1.130583	0.865098	1.030509	0.997151	0.938844
Case D-Clone 2	0.771132	1.015123	0.642047	0.644748	0.910343	0.804618	0.999325	1.264515	0.864164	0.966514	0.766406	1.139076
Case E-Clone 1	0.942816	0.86502	0.989252	0.748859	0.990243	0.684371	1.085452	1.061314	1.146683	0.882363	0.897512	0.926676
Case E-Clone 2	1.010119	0.952611	0.657507	0.653947	0.814718	0.920742	1.199021	1.038338	1.056855	0.749911	0.920119	1.035757

Raw gel images

Figure 1a

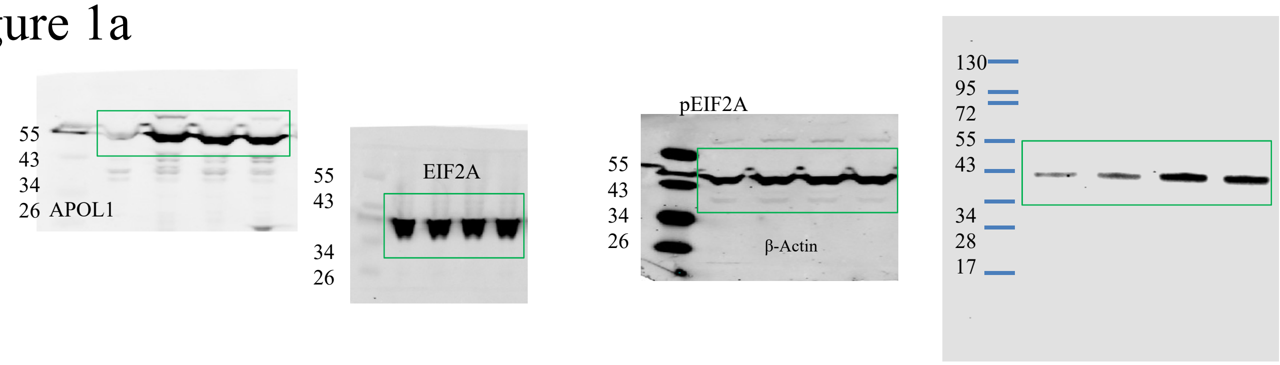

Figure 1b

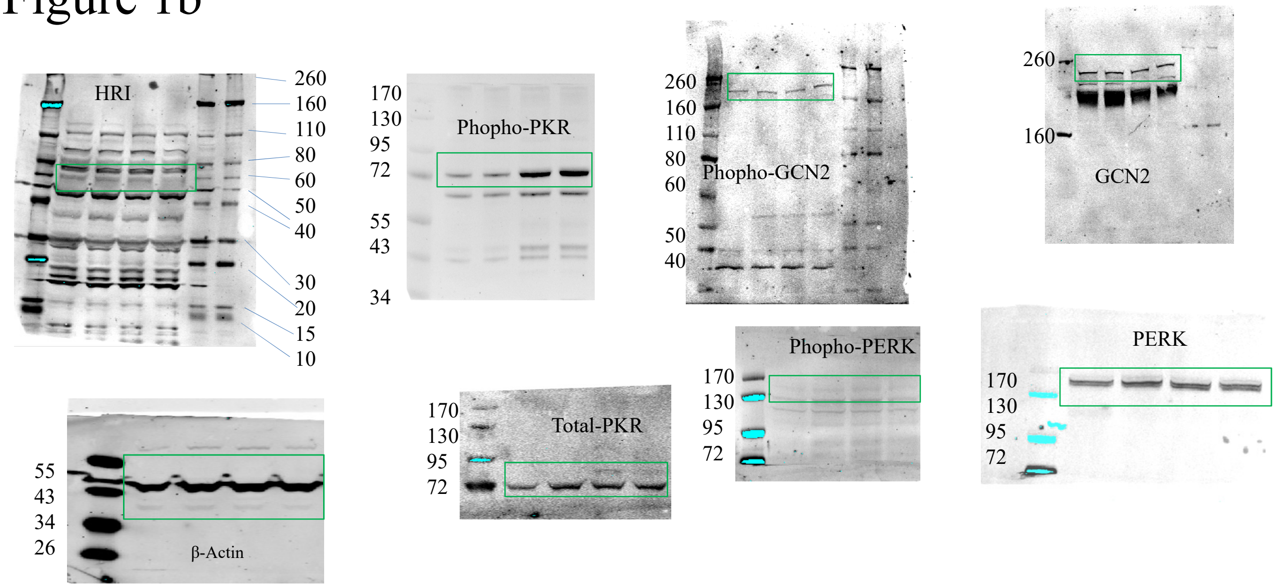

Figure 1c

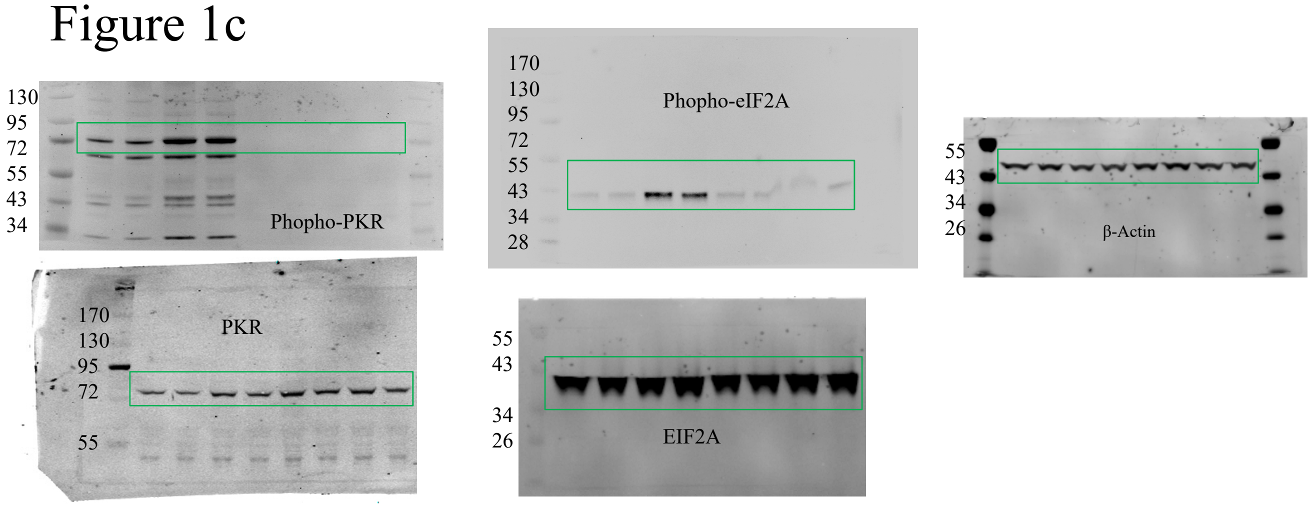

Figure 2a

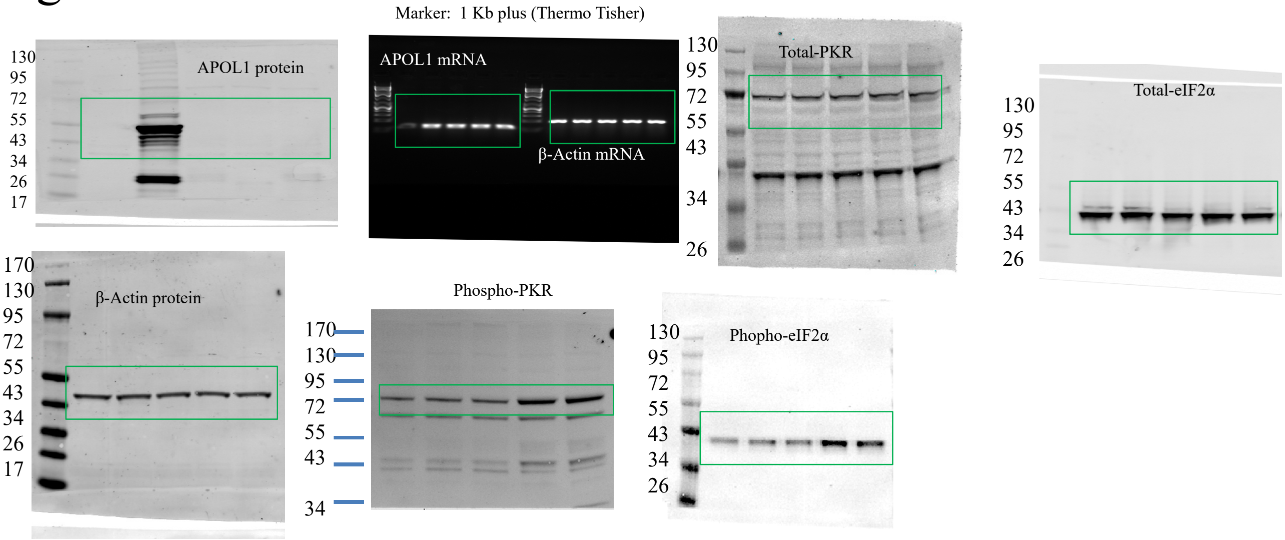

Figure 2b

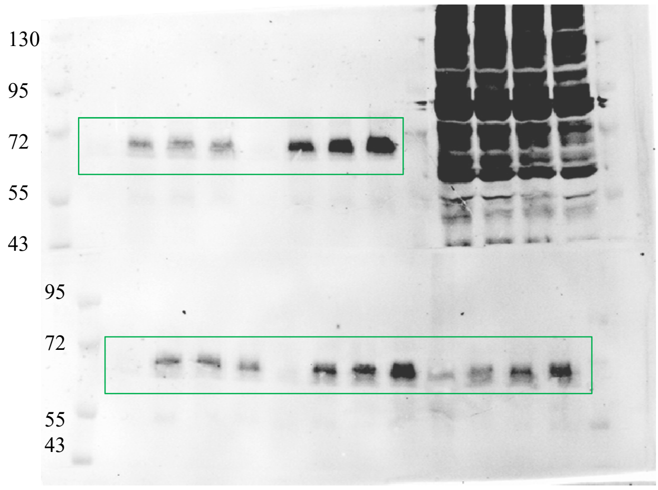

Figure 3c

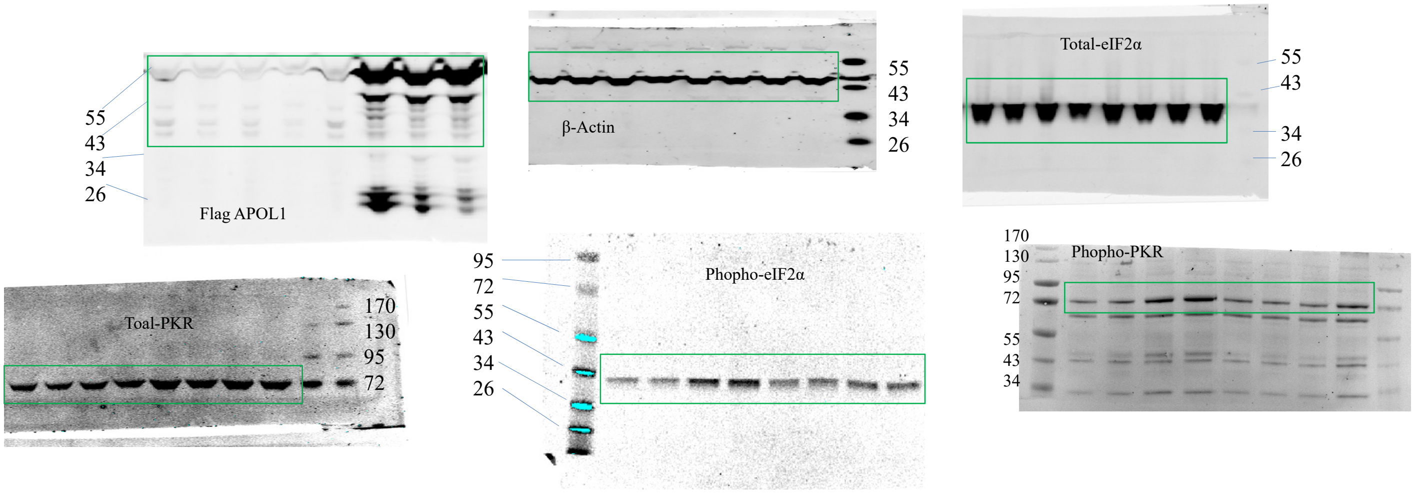

Supplementary Figure 2d

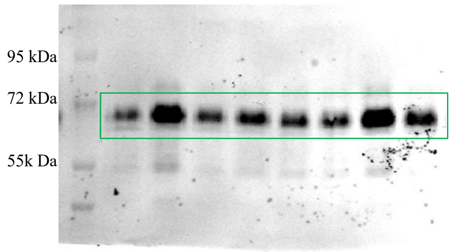

Supplementary Figure 2e

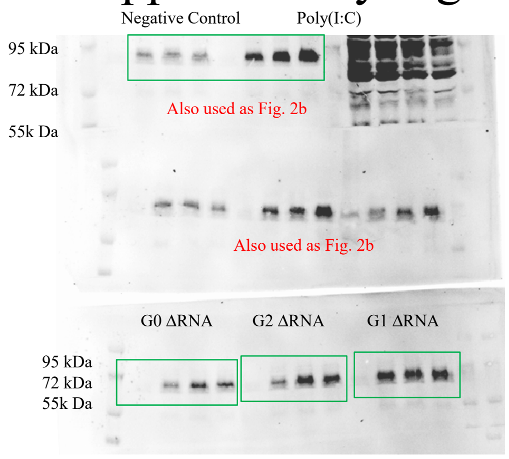

Supplementary Figure 4d

Left 5 lanes were also used as Extended Data Figure 1e

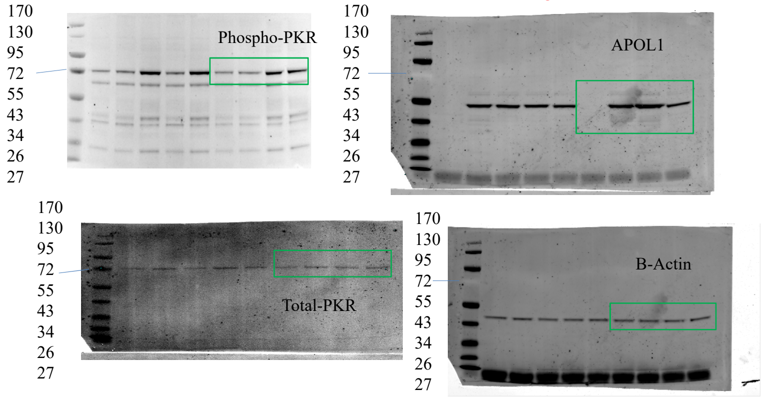

Supplementary Figure 5c

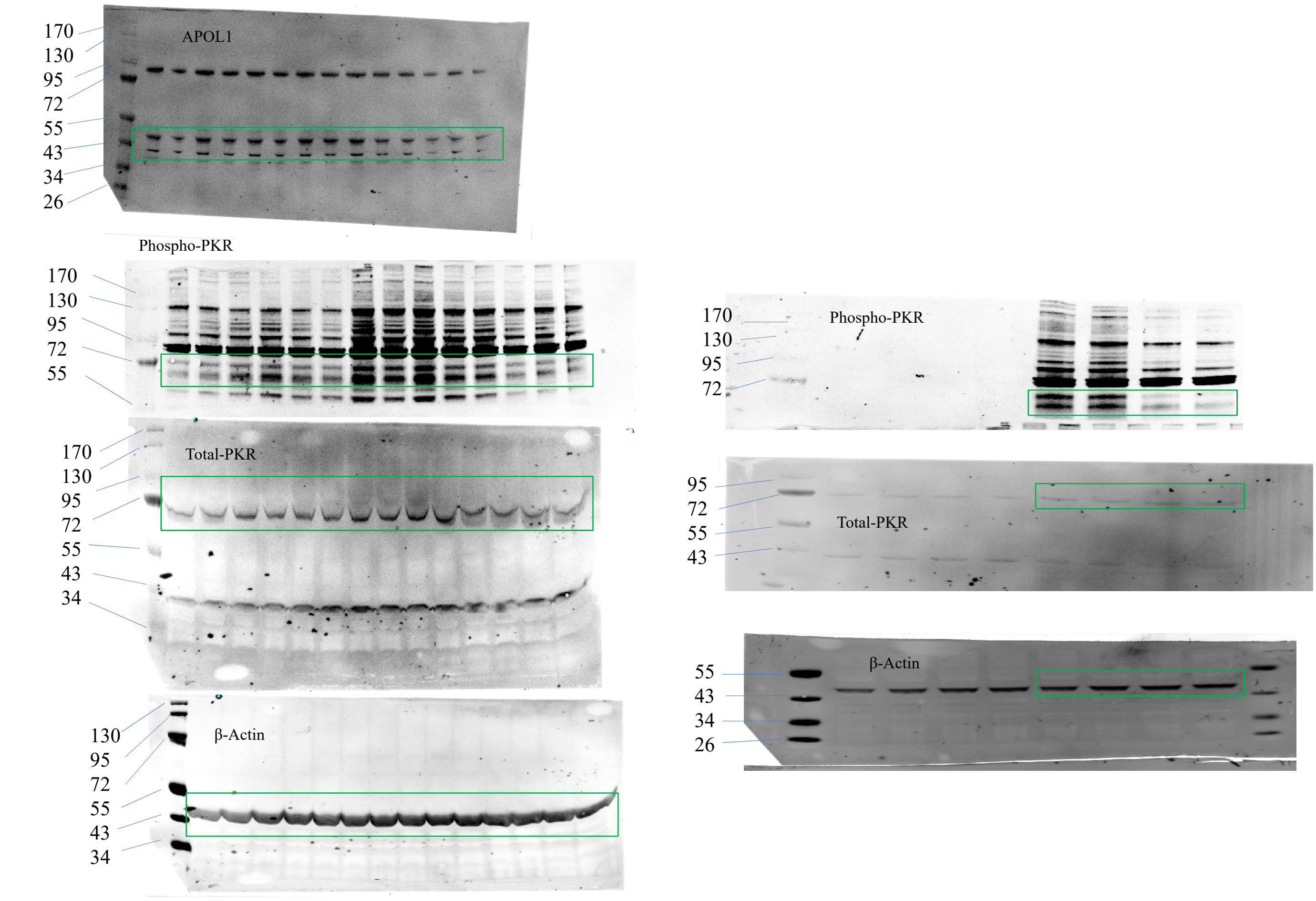

Supplementary Figure 6b

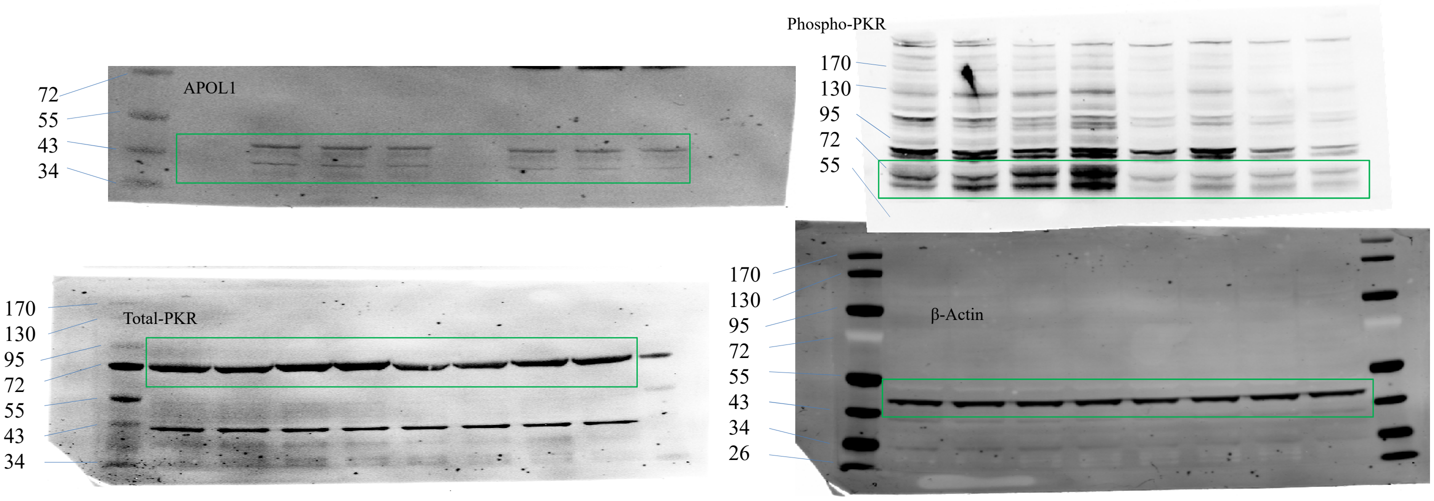

Supplementary Figure 6f

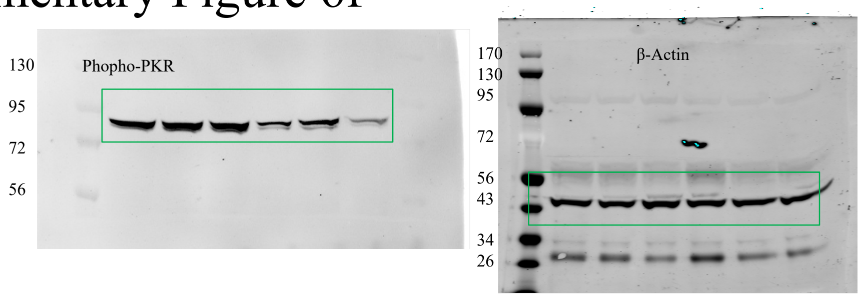

Supplementary Figure 7a

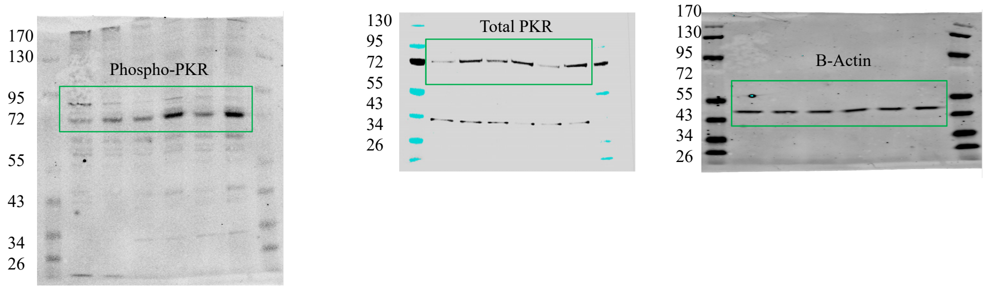

Supplementary Figure 7b

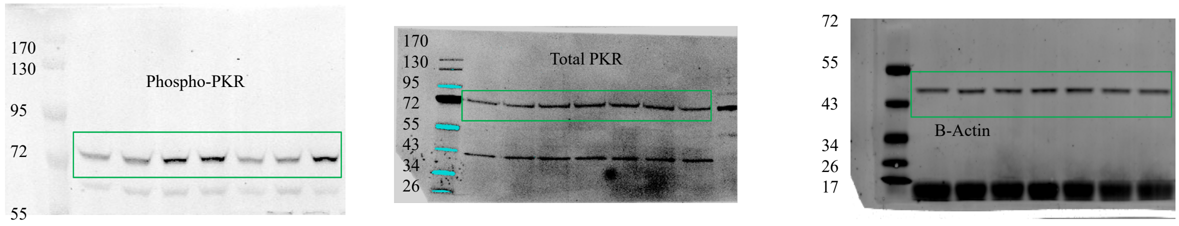

Supplementary Figure 8b

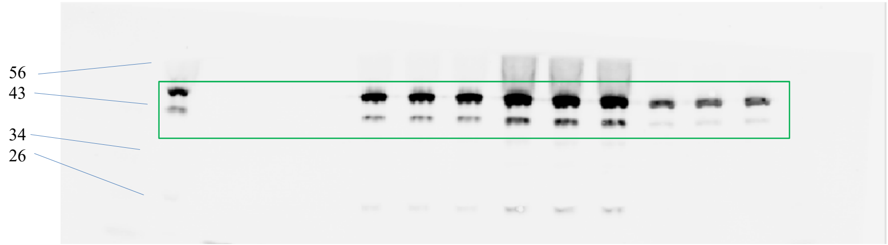

Supplementary Figure 9

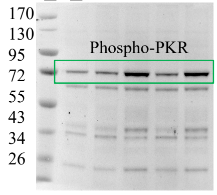

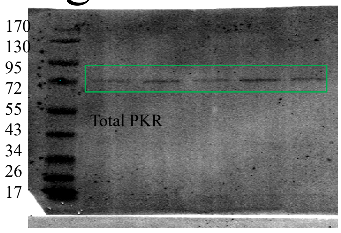

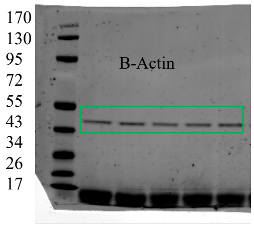

Supplementary Figure 10

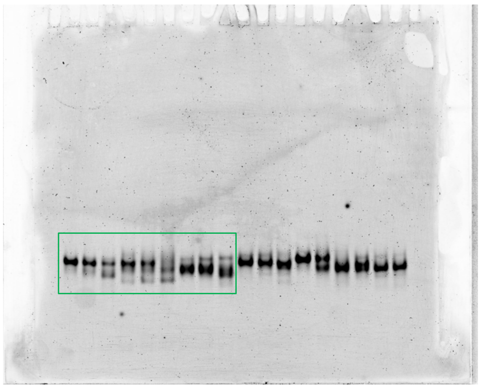

Supplement: Supplementary file 1 — Supplementary file [file 42003_2018_188_MOESM1_ESM.pdf]
